# Directed mutagenesis of large multi-subunit protein complexes by plasmid sub-fragmentation

**DOI:** 10.1038/s41598-026-53234-8

**Published:** 2026-05-25

**Authors:** Adel Beghiah, Ville R. I. Kaila

**Affiliations:** The Arrhenius Laboratories for Natural Sciences, Department of Biochemistry and Biophysics, Stockholm University, SE-106 91 Stockholm, Sweden

**Keywords:** Biochemistry, Biological techniques, Biotechnology, Computational biology and bioinformatics, Molecular biology

## Abstract

Site-direct mutagenesis provides a basis for functional studies of proteins. Yet, despite many available techniques, mutagenesis of large protein complexes can be tedious and highly challenging, with the replication fidelity and long-range amplification limiting the process. Here, we develop a method for site-directed mutagenesis of large protein complexes based on sub-fragmentation of its coding sequence that generates libraries of shorter sequences, where the DNA polymerase can accurately catalyse long-range elongation. Using the *nuo* genes encoding for the 0.5 MDa *E. coli* Complex I, we successfully dissect the 15.1 kb long DNA sequence into fragments of 900 bp with partially overlapping regions and unique primer sequences, creating a plasmid library that can be amplified by regular DNA polymerases. We showcase the method by sub-cloning the *E. coli* Complex I sequence into 20 fragments from a large pBAD expression vector (21.3 kb). The method shows a high success rate of introduced mutations, and an efficient Gibson assembly of the mutated fragments into the expression vector, enabling the accurate introduction of point-mutations into the large pBAD vector. We suggest that the plasmid sub-fragmentation provides an efficient method for mutagenesis of multi-subunit protein complexes with several homologous subunits, such as the respiratory Complex I, advancing mechanistic studies of large bioenergetics protein complexes.

**Supplementary Information:**

The online version contains supplementary material available at 10.1038/s41598-026-53234-8.

## Introduction

Mutagenesis is a key technique in molecular biology, where substitutions are introduced into a DNA sequence encoding for the amino acid sequence of a protein of interest. Mutagenesis provides the basis for probing structure–function relationships in proteins and elucidate their mechanistic principles. Site-directed mutagenesis, which created the field of protein engineering, was introduced in the late 1970s^1^ that together with the development of the polymerase chain reaction (PCR)^2^ allows for the insertion, deletion or substitution of nucleotides in a sequence of interest. Amongst the various techniques developed, QuickChange^3^ has become one of the most commonly used methods that relies on the introduction of a pair of complementary oligonucleotides in a primer extension process, with one or several mutations of interest. The plasmid is then amplified using a high-fidelity polymerase such as Pfu^3^.

QuickChange and related techniques such as side-directed Ligase Independent Mutagenesis (SLIM)^4^ and the overlapping PCR method^5^, are often less efficient for larger protein complexes (> 15 kb) as the long-range elongation accuracy is often limited to 15 kb for most common polymerases, such as Taq, Pfu, and the Q5 DNA polymerase. The rise of the CRISPR-Cas9^6^ technology significantly enhanced the feasibility of chromosomal editing, and could also be applicable to large plasmid mutagenesis although costs required to apply these methods still remain a major limiting factor in many practical applications. Others techniques, applicable on whole genomes or bacterial artificial chromosomes^7,8^, can also be used, such as homologous recombination, which is based on the insertion of a readily mutated fragment including blunt ends digested by an exonuclease to create single strand (ss) DNA ends. The ssDNA is then recognised by another polymerase allowing the annealing and formation of the targeted recombinant DNA.

**Fig. 1:**
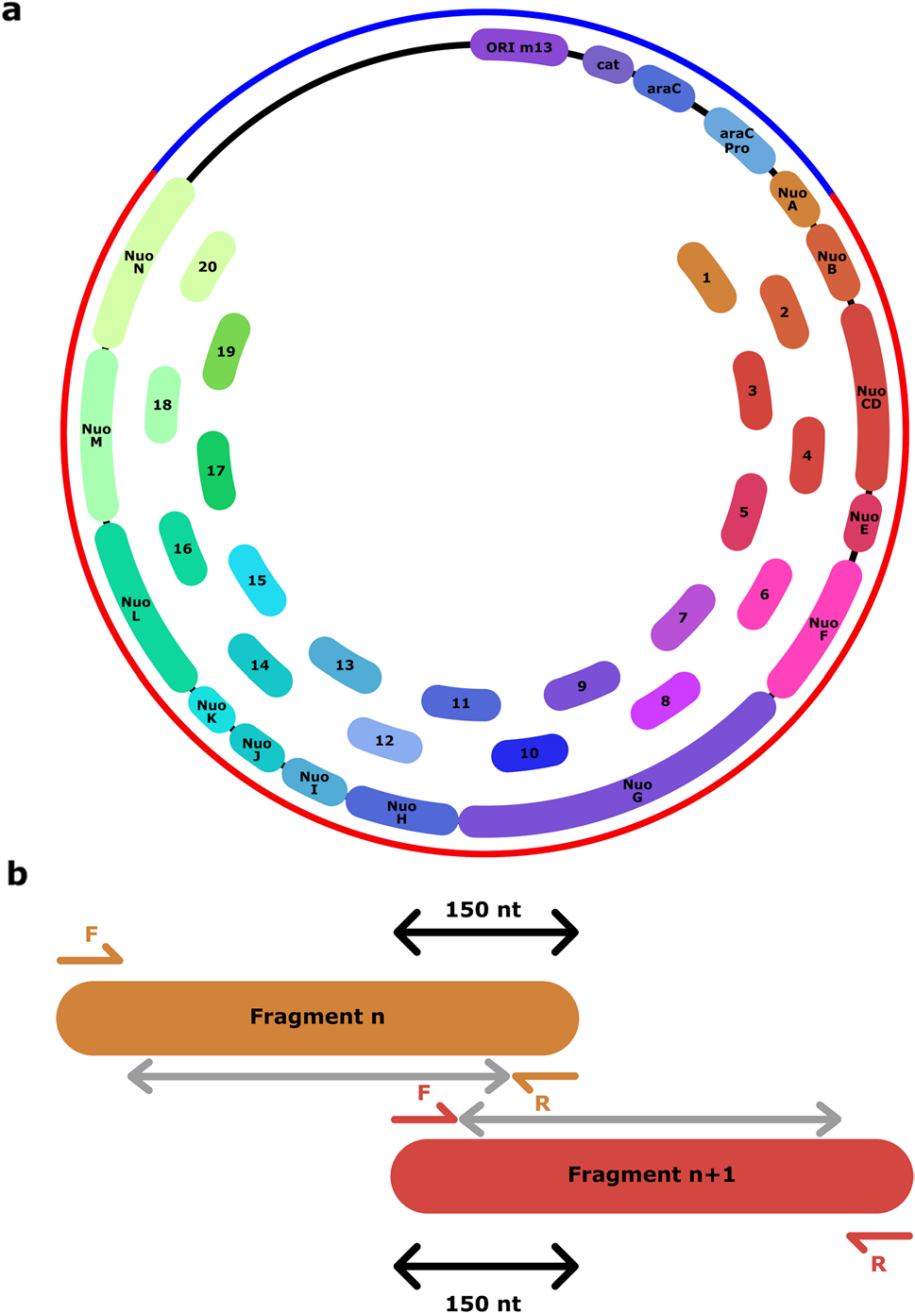
Sub-fragmentation of the pBAD_*nuo*_ construct. (**a**) Map of the sequence coding for CI segmented into 19 fragments of 900 bp and one fragment of 950 bp. The outer circle represents the pBAD_*nuo*_ plasmid, with the DNA sequence encoding for *Ec*CI in red, and in blue the pBAD vector including the resistance cassette for chloramphenicol (cat), origin of replication M13, and gene coding for the protein AraC regulating the promoter araC. (**b**) Overlapping 150 nt regions between fragment *n* and *n* + 1. The overlapping region was designed so the forward *n* + 1 primer is part of the first 75 nt of the overlapping region, and the reverse *n* primer is part of the last 75 nt of the overlapping region (detailed position in Supplementary Table S1). The grey arrow corresponds to the region where mutagenesis can be achieved in fragment *n* or *n* + 1 according to the nucleotide position.

Mutagenesis of large protein complexes provides an essential methodology to establish functionally relevant sites to experimentally testing mechanistic hypotheses. In this regard, mechanistic studies of the respiratory Complex I (NADH:ubiquinone oxidoreductase) impose significant challenges to modern biochemical approaches due to its large (0.5–1 MDa) membrane-bound structure, comprising up to 9000 amino acids and several cofactors. Complex I is a central protein of aerobic respiratory chains responsible for oxidative phosphorylation. It catalyses the oxidation of nicotinamide adenine dinucleotide (NADH), which initiates electron transfer along the hydrophilic domain of the protein towards ubiquinone (Q_10_), while the Q_10_ reduction triggers proton pumping across the 200 Å wide membrane domain^9–13^. Despite decades of structural, genetic, and functional studies^10,11,14–20^, the mechanism of Complex I remains elusive and highly debated, in part due to challenges in generating functionally relevant mutations. Mutagenesis experiments have been essential in the identification of important coupling sites in Complex I^20–29^, although the large plasmid size is limiting the efficiency of the genetic methods used. The *E. coli* Complex I (*Ec*CI) is a 0.5 MDa enzyme complex, encoded by a 15.1 kb DNA sequence, which renders it particularly challenging for traditional mutagenesis approaches. For example, the commonly used DNA polymerases such as Taq and the Pfu polymerase, have limitation in both fidelity and long-range amplification, restricted to about 5 kb. Many polymerases also feature a 3′ → 5′ exonuclease domain responsible for removing the incorrectly incorporated nucleotide (nt) during DNA replication, also known as proofreading^30^. Although, this enhances the replication fidelity, a suitable polymerase is required for a reliable amplification of large plasmids. Molecular biology of *Ec*CI has been achieved using the pBAD vector, commonly used in overexpression of membrane proteins. This vector contains a promoter allowing for the tight regulation of protein expression, while the promoter is regulated by the AraC protein (encoded by the *araC* gene), which activates transcription upon binding to arabinose. The pBAD vector also contains a selectable marker, cloning sites, and the f1 bacteriophage origin of replication sequence determining the copy number of the plasmid (Fig. 1). Previously, Complex I variants have been generated using, *e.g.*, QuickChange kits^26,31,32^. However, the large-scale screening of residue substitutions requires more efficient approaches than those currently available. Moreover, the systematic introduction of mutations into unique sites can also be hampered by the homologous subunits NuoN/M/L in Complex I that have a 20–30% sequence similarity.

With aims to enhance the efficiency and reliability of mutagenesis of large (> 20 kb) DNA constructs, we proposed here a *plasmid sub-fragmentation* (PSF) strategy that relies on creating a vector library, comprising sub-fragments of a large plasmid construct. As opposed to other methods allowing for modular cloning (*e.g.*, Golden Gate assembly^33,34^), our approach does not rely on restriction enzyme limited only to their recognition sites to generate fragments. Moreover, the fragment size generated by the PSF strategy is highly controlled through the primer pairs, specifically designed for each fragment, which supports a high-quality reading (< 1100 nucleotides based on Sanger sequencing, Eurofins Genomics), in contrast to, *e.g.*, the Gateway method that can introduce scars to the newly added fragments^35,36^. To this end, we subclone the generated sub-fragments into a pUC19_∆LacZα_ cloning vector, where the directed mutagenesis can be efficiently achieved using regular polymerases. This drastically decreases the error rate, and increases the feasibility of generating precise site-directed point mutation (see Results). After sequencing, the mutated fragments are inserted back into the original plasmid using Gibson assembly.

## Results

### Fragment design

Mutation of residues in large protein complexes, such as the OXPHOS complexes of respiratory chains, can be highly challenging and hampered by the poor success rate of incorporating substitutions, resulting in low yields. To overcome these bottlenecks, we applied the proposed PSF approach to introduce substitutions into the pBAD_*nuo*_ vector. To this end, we dissected the original plasmid into multiple, partially overlapping sub-fragments that allows the DNA polymerase to operate within its long-range amplification and fidelity limits (5–15 kb for Taq, Phusion, and Q5 DNA polymerase). Here, the *E. coli* Complex I (*Ec*CI) serves as a model of a large protein assembly to introduce single point substitutions, although the approach is generally applicable for any protein complex that can be partitioned into overlapping sequences.

The intact pBAD_*nuo*_ construct^37^, comprises 21,360 base pairs, including the sequences coding for the 13 subunits of the *Ec*CI (15,180 bp), a chloramphenicol resistance cassette, as well as a His_6_-affinity tag on NuoF, the subunit responsible for NADH oxidation and is thus beyond the fidelity and long-range elongation threshold of commonly used DNA polymerases. To test the method, we created a fragment library of the pBAD_*nuo*_ vector (Fig. 1a) by dissecting the DNA sequence encoding for the *Ec*CI into 19 fragments, 900 bp each, and one fragment comprising 950 bp (Fig. 1a, Supplementary Table S1). Each fragment *n* comprised 150 bp overlapping sequences with the adjacent fragments (*n − *1 and *n* + 1) (Fig. 1b). Unique amplification primer pairs were designed for each fragment, such that the primer binds to the last 75 nt (reverse primer) and the first 75 nt of the overlapping region (forward primer, Fig. 1b, Table 1). This design allows for the introduction of substitutions at any position of the plasmid.

**Table 1:** List of primers used for PCR.

Primer name (5′ → 3′)	Forward	Reverse
pUC19_linearisation	AGCTGTTTCCTGTGTGAAATTGTTATCCG	TTAAGCCAGCCCCGACACCC
pUC19_sequencing	TGTAAGCGGATGCCGGGAGCAGACAAGC	ACACTTTATGCTTCCGGCTCG
Fragment_12_linearisation	TCGGGGCTGGCTTAACGGGCTTTCCTTGATGGGCG	ACACAGGAAACAGCTATCCTGGCGCTTGTATTCCCCC
pUC19-12_linearisation	GGCTGGTCAAACGGGTGACGGTGACATACCGCCACG	GCTACGAAGTGTTCCTCGGGCTTTCCTTGATGGGCG
pBAD_Δ12__linearisation	GGGAATACAAGCGCCAGGATCTGGTTTACGAGAAAGAGGA	CGCCCATCAAGGAAAGCCCGAGGAACACTTCGTAGCTCAG
Fragment_1_linearisation	TCGGGGCTGGCTTAAGCTAGCGAATTCGAGCTCGGTACCC	ACACAGGAAACAGCTACCCATTTTGGTTCCAGCATCTGGTC
pUC19-1_linearisation	TACCCGTTTTTTTGGGCTAGCGAATTCGAGCTCGGTACCC	GCACCCATTGAGATAACCCATTTTGGTTCCAGCATCTGGTC
pBAD_Δ1__linearisation	ATGCTGGAACCAAAATGGGTTATCTCAATGGGTGCCTGTG	CCGAGCTCGAATTCGCTAGCCCAAAAAAACGGGTATGGAG
pUC19-12_E216Q	GTTTGACCAGCCGCAAGCCGAG	CGGCTGGTCAAACGGGTGACG
pUC19-12_E218Q	GGAAGCCCAGCAGGAACTGG	GGCTTCCGGCTGGTCAAACGG
pUC19-12_E218R	GGAAGCCCGGCAGGAACTGG	GGCTTCCGGCTGGTCAAACGG
pUC19-12_E241Q	GTTCTTCGTGGGTCAGTACATCGGGATTG	ACCCACGAAGAACAGACCGAACTTC
pUC19-12_E241A	GTTCTTCGTGGGTGCGTACATCGGGATTG	ACCCACGAAGAACAGACCGAACTTC
pUC19-13_I63M	GTCTACGCGGGTGCCATGATGGTGCTGTTCGTGTTCGTGGTGATG	GGCACCCGCGTAGACGATAATTTCC
pUC19-13_I63A	GTCTACGCGGGTGCCGCTATGGTGCTGTTCGTGTTCGTGGTGATG	GGCACCCGCGTAGACGATAATTTCC
pUC19-1_D79A	TTCTTCGTTATCTTCGCCGTTGAAGCGCTG	GAAGATAACGAAGAACATGGCCACCAGATAAAAC
pUC19-1_E51A	AAAAACGTGCCGTTTGCATCCGGTATCGACTCG	AAACGGCACGTTTTTCGACCTCGCG

### Generation of a pUC19 sub-cloning library

**Fig. 2:**
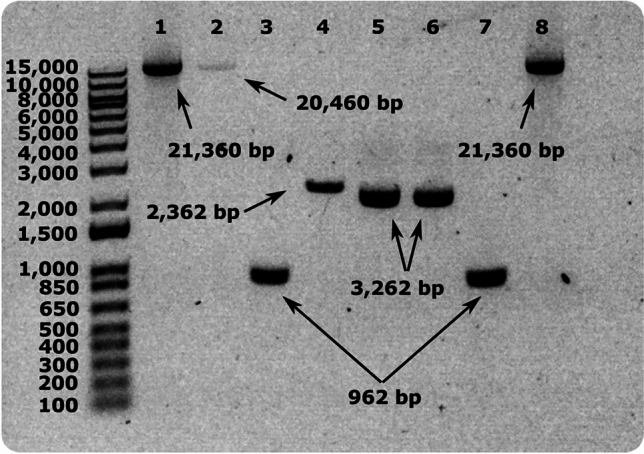
Agarose gel showing (1) the pBAD_nuo_ intact plasmid, (2) the pBAD_nuo_ linearised plasmid excluding fragment 12, (3) the WT fragment 12, (4) the linearised pUC19_ΔlacZ_, (5) pUC19-12, (6) pUC19-12-E216Q^H^, (7) fragment 12 of E216Q^H^, and (8) the pBADnuo-E216Q^H^.

**Fig. 3:**
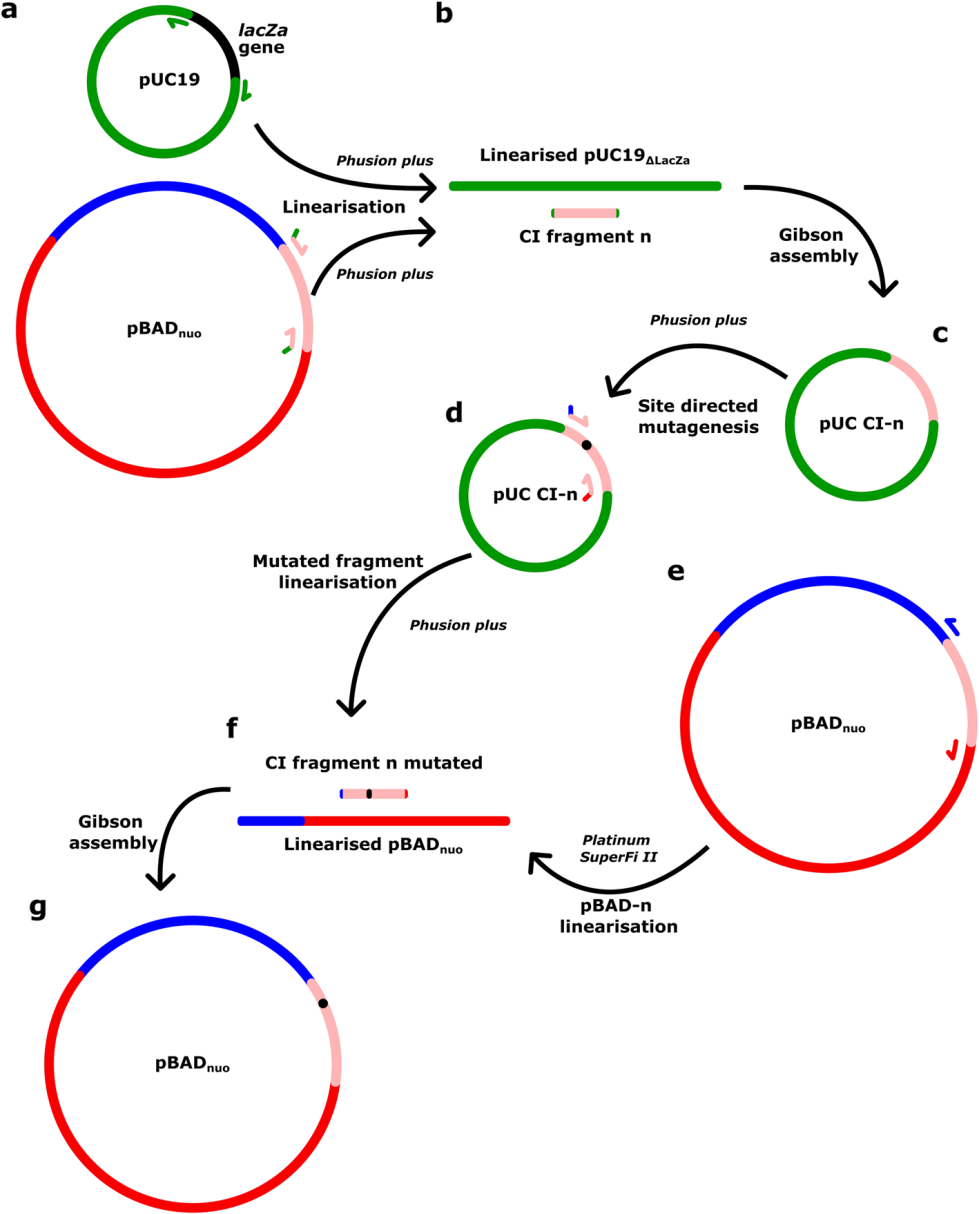
Principles of the plasmid sub-fragmentation approach. (**a**) Linearisation by the Phusion plus DNA polymerase of the pUC19 vector, excluding the gene lacZα and the fragment *n* of interest, which encodes for a targeted region of *Ec*CI. Forward and reverse primers are represented by arrows. (**b**) CI fragment *n* including 15 nt complementary to the linearised product pUC19_ΔLacZa_ for (**c**) Gibson assembly creating the new construct pUC CI-n. The pUC CI-n construct is used for (**d**) site-directed mutagenesis, with point mutation represented by a black dot. Linearisation primers, represented by pink arrows, are designed to be 20–30 nt long, together with a 15 nt sequence complementary to both end of the (**e**) pBAD_*nuo*_ linearised product (blue and red). (**f**) Linearised and the mutated fragment of pBAD_*nuo*_ are incubated together for Gibson assembly, resulting in (**g**) the reassembled pBAD_*nuo*_ construct, with the substitutions.

The fragments were amplified from the WT-pBAD_*nuo*_ construct using primers generated using SnapGene (Table 1). In parallel, the pUC19 cloning vector was amplified by excluding the *lacZa* gene, (Fig. 2 lane 4, Fig. 3a), replaced by the fragment encoding for a targeted region of *Ec*CI (Fig. 3b,c). To do so, we designed 20–30 nt long primers for the fragment amplification and pUC19_ΔLacZa_ linearisation (amplification primers) (Table 1). A common complementary sequence for the pUC19_ΔlacZa_ of 15 nt was added on the 5′ end of each forward primer (TCGGGGCTGGCTTAA 5′ → 3′) and each reverse primer (ACACAGGAAACAGCT 5′ → 3′) used for fragment linearisation (Fig. 3a, bottom inset in green). The common complementary sequence included on the primer pairs were designed for the different CI fragment amplifications to facilitate the Gibson assembly and formation of the new pUC19 CI-*n* constructs (Fig. 3b in green). PCR reactions for pUC19_ΔLacZa_ and fragment linearisation were performed individually using the Phusion plus DNA polymerase (Thermofisher), resulting in the linear DNA products (Fig. 3b). The cloning results of each step were characterised by agarose gel electrophoresis (Fig. 2, lanes 3 and 4, Supplementary Fig. S1) prior to DpnI digestion, performed for the removal of the parental DNA template used for the PCR reaction. The *n* fragments and the pUC19 linearised product were used for Gibson assembly to attach both linearised PCR products ( Figs. 2 lane 5, 3b,c). A similar sub-cloning approach was applied on all 20 fragments extracted from the pBAD_*nuo*_ plasmid (Supplementary Fig. S2).

### Site-directed mutagenesis of the pUC19 cloning vectors

Variants of Complex I were created within the cloning vector pUC19, containing the 900 bp DNA fragment (pUC CI-*n*) inserted by Gibson assembly (Fig. 3d). To this end, we tested the mutagenesis approach for three fragments, as a *proof-of-concept*, within the 20-fragment library, encoding for a region of the NuoH, NuoJ and NuoA subunits, respectively, by introducing the E216Q^H^, E218Q^H^, E216R^H^, E241Q^H^, E241A^H^, I63A^J^, I63M^J^, E51A^A^, and D79A^A^ single point mutations, based on key functional residues identified in our previous mechanistic studies^38^. Primers were designed (Table 1) for site directed mutagenesis within the pUC19 CI-*n* vector with a PCR reaction using the Phusion plus DNA polymerase (Thermofisher). The outcome of the PCR reaction was verified by agarose gel (lane 6, Figs. 2, 3d) and sequencing (Supplementary Table S2, Supplementary Table S3).

### Back-insertion of the mutated fragment into pBAD

In order to incorporate the mutated fragment into the original construct, the pBAD_*nuo*_ plasmid was linearised by amplifying the vector without the fragment of interest (lane 2, Figs. 2, 3e) using the Platinum SuperFi II DNA polymerase (Thermofisher) (see Method), together with linearisation of the mutated fragment in pUC19 CI-*n* (lane 7 of Figs. 2, 3d). The designed primers (20–30 nt long) used for the mutated fragment linearisation included 15 additional nucleotides added to each primer 5′ end (Fig. 3d) to be complementary to the pBAD linearised ends in order to facilitate the fragment incorporation. Both products were incubated together for Gibson assembly, and verified by agarose gel (lane 8 of Figs. 2, 3f,g).

### Efficiency of PSF mutagenesis

The efficiency of the PSF mutagenesis strategy was assessed by counting the positive clones obtained from sequencing results. The fragment linearisation and insertion into the pUC19_ΔLacZa_ vector led to 100% incorporation of the fragment tested, without any error inserted to the sequences of interest (Supplementary Fig. S4a). A total of nine different mutations were then tested in the vectors pUC19-1, pUC19-12, and pUC19-13. Each mutation led to a success rate range of 33–100% (Supplementary Fig. S4b). Overall, for the 22 colonies tested, 13 colonies produced positive clones, with 100% conservation of the sequence except for the substitution of the targeted codon. The mutated fragment incorporation into the pBAD vector was less efficient, especially for one mutant (I63M^J^) resulting in only one positive colony out of 7 screened, *i.e.,* 14% percent efficiency (Supplementary Fig. S4c). Other mutants gave a positive colony in 25–100% of the tested cases. Overall, for 25 colonies screened, 9 were positive (36%), considering the challenging I63M^J^ mutations, against 44% of positive clones without I63M^J^.

### pUC19 and pBADnuo plasmid sequencing results

The assembled pBAD_*nuo*_ plasmid was transformed into the DH5α Δ*nuo* strain for DNA isolation. The purified DNA was prepared and sent for sequencing (see *Methods*) to confirm that the targeted mutation is the only substitution observed within the 21,360 base pairs sequence (Supplementary Fig. S3). The mutations of interest in the NuoH, NuoJ and NuoA subunits, present at the interface between the membrane and the hydrophilic domain of Complex I were incorporated by substituting one or two nucleotides from the corresponding codon, in the forward primer (Table 1), used for site-directed mutagenesis in the corresponding pUC19 CI-*n* cloning vector. When placed back to the pBAD_nuo_ plasmid, the substitution was first confirmed to be present within the inserted fragment, followed by whole plasmid sequencing (Macrogen Europe). Positive clones confirmed by sequencing (Eurofins Genomics) led to 100% success rate in whole plasmid sequencing as the only mutation observed in all samples are the nucleotides substituted within the targeted codons (Supplementary Fig. S5, Supplementary Table S2). The translated sequence also showed that the amino acid substitution was effective and exclusive (Supplementary Fig. S3, Supplementary Table S3), as no other unspecific mutation was observed, confirming the reliability of this approach using both polymerases together with suitable vectors, in respect to the high fidelity and long-range amplification properties (Supplementary Fig. S6).

### Overexpression and assembly of complex I variants

**Fig. 4:**
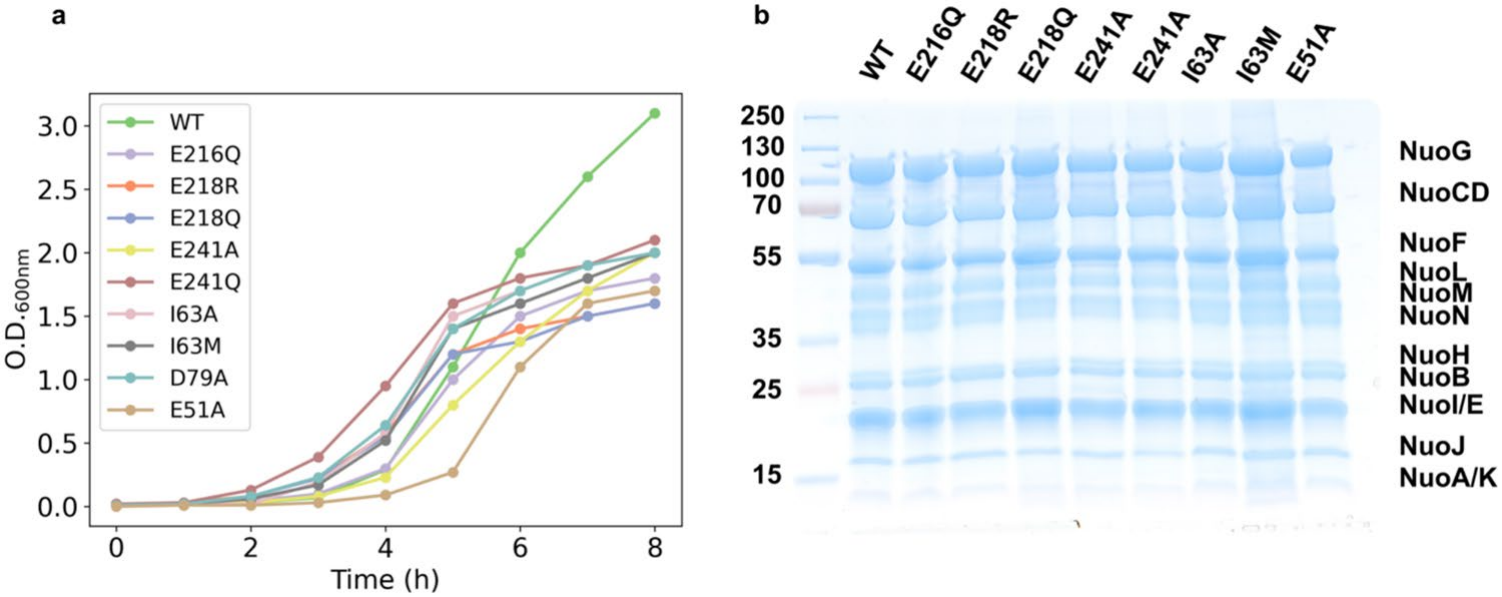
Expression of PSF-generated Complex I variants. (**a**) BW25113_*Δndh nuo::nptII_FRT*_ growth test expression of *E. coli* WT Complex I and variants. (**b**) SDS-PAGE gel showing all Complex I subunits for the WT and purified variants.

We next performed growth tests to assess the ability of each PSF-generated Complex I variant to allow the transformed strain to grow (Fig. 4a). Each mutant resulted in fully grown cells with OD_600_ = 1.5 within 5–8 h, whereas the WT Complex I was grown until OD_600_ = 3.0 over 8 h (Fig. 4a). Moreover, the purified protein of each variant showed the presence of all subunits on an SDS-PAGE gel (Fig. 4b), and validating that application of the PSF method results in correctly expressed Complex I.

## Discussion

Directed mutagenesis is a fundamental tool in molecular biology that provides the basis for elucidating structure–function relationships. The site-directed mutagenesis relies on a pair of oligonucleotides containing a mutation of interest to generate protein variants via PCR, which can then be used for the functional characterization. However, many polymerases used for the PCR have limited high replication accuracy, while the application of polymerases beyond their amplification range, typically up 15 kb, reduces the overall success of the mutagenesis experiment. High fidelity amplification becomes particularly challenging for plasmids encoding for large protein complexes, such as the respiratory Complex I, encoded by the 21.3 kb pBAD_*nuo*_ plasmid, which extends beyond the amplification range of most polymerases. To tackle this challenge, we introduced here mutations into the large pBAD_*nuo*_ plasmid by the proposed PSF approach.

Previous mutagenesis studies of Complex I have relied on methods such as QuickChange, and thus limits the amplification of large protein construct^14,26,31,39,40^. In this regard, the proposed PSF approach allows for the selection of suitable polymerases and adjustments of the plasmid model, based on the properties of the polymerase. The pUC19 CI-*n* construct (3.2 kb), built based on fragments with *n* = 900 bp, allows the polymerase to operate within high-fidelity limit, thus enhancing the efficiency of the site-directed mutagenesis experiment. To achieve this, we employed here the Phusion Plus DNA polymerase (Thermofisher), which enables extended template PCR with an upper limit of 10 kb on genomic DNA and up to 20 kb on low complexity DNA (Thermofisher) (> 100 × fidelity in comparison to the Taq DNA polymerase), allowing for the plasmid amplification with a low error rate and accurate incorporation of substitution(s). The pBAD_*nuo*_ vector template amplification was performed using the high-fidelity Platinum SuperFi II DNA polymerase (Thermofisher), which has a higher long-range elongation, enabling amplification of up to 20 kb for human gDNA and up to 40 kb for bacterial gDNA (> 300 × fidelity relative to the Taq DNA polymerase).

Complex I from *P. denitrificans (PdCI)* has also been shown to provide a tractable model system to study the function of Complex I^27^. Expression of the *PdCI* relies on the an inducible pLMB509 vector^41^, which differs in many regards from our current pBAD_*nuo*_ vector. While conjugation of pBAD into *P. denitrificans* could in principle be possible, the pBAD_*nuo*_ vector lacks an origin of transfer (oriT), while the process is also likely to require an exchange to another inducible system (such as rhamnose) to support the induction of CI expression. Nevertheless, we suggest that the general PSF approach could be applied also to generate fragment libraries on other plasmids and thus locally generate mutations in large plasmids.

Notably, the sub-fragmentation approach is also applicable for significantly larger plasmids that the current 21.3 kb construct used here and can be achieved by further fragmentation of the sequence to smaller sub-fragment. In addition to allowing the polymerase to operate within its optimal amplification range, the sub-fragmentation provides benefits for mutagenesis of multi-subunits protein complexes comprising several homologous subunits such as the NuoN/M/L subunits of the *Ec*CI, which have a 20–30% sequence similarity, and where, *e.g.*, QuickChange may introduce non-specific substitutions, while in the sub-fragmentation approach, these subunits reside in individual fragments (Supplementary Fig. S2).

Taken together, by addressing limitations of traditional techniques, we show here that the plasmid sub-fragmentation (PSF) approach provides an efficient method for site-directed mutagenesis of large multi-subunits membrane protein, allowing for a high flexibility in the fragment design.

## Material and methods

### Plasmid, DNA linearisation and mutagenesis

The pBAD_*nuo*_ plasmid^37^ was used as a template for the DNA sub-fragmentation (Fig. 1), and the pUC19 (NEB) as a cloning vector for inserting the fragments. pBAD_*nuo*_ was linearised (see PCR conditions in Supplementary Table S3, S4) using the Platinum SuperFi II DNA polymerase (Thermofisher), and the pUC19 linearised (see PCR conditions in Supplementary Table S3, S4) using the Phusion plus DNA polymerase (ThermoFisher). The pUC19 vector and the 900 base pairs fragments (see PCR conditions in Suppementary Table S3, S4), were created using PCR method, by engineering primers (Table 1) designed on SnapGene (Thermofisher). Single point mutations (see PCR conditions in Supplementary Table S3, S4) were incorporated using the Phusion plus DNA polymerase (Thermofisher). The template DNA was removed by DpnI digestion prior to transformation in the *E. coli* DH5α_∆nuo_ cloning strain (F- Φ80*lac*Z*Δ*M15 *Δ*(*lac*ZY A-*arg*F) U169 *recA1 endA1 hsdR17* (rk- mk +) *gal- phoA supE44* λ- *thi-1 gyrA96 relA1*, *Δnuo*)^42^.

### Gibson assembly and transformation

Linearised products of pBAD_*nuo*_ and pUC19 were fused with the Complex I fragment using Gibson assembly^43^ (New England Biolabs Gibson Assembly mix, 1:2 plasmid:insert ratio). The resulting products were transformed into the DH5α_∆nuo_ cloning strain and plated in presence of chloramphenicol or ampicillin, prior to incubation overnight at 37 °C. The plasmid purification was performed using the GeneJET Plasmid Miniprep kit (Thermo Scientific).

### Sequencing

Fragment insertion and substitutions for the variants were confirmed by sequencing (Eurofins genomics, Uppsala, Sweden). The whole-plasmid sequencing was performed by the Nanopore Technology service (Macrogen Europe).

### Protein expression and purification

The pBAD_*nuo*_ plasmid was used to transform the BW25113_*Δndh nuo::nptII_FRT*_ expression strain^44^ for overexpression of WT or Complex I mutants into autoinduction media (Supplementary Table S4). Cells were collected, resuspended in cell resuspension buffer (Table S4) and lysed in presence of 0.1 mg mL^−1^ DNase, and 0.5 mM protease inhibitor (AEBSF, Sigma Aldrich), using an Emulsiflex (15,000 psi). Cell debris were removed by centrifuging the sample at 20,000 g for 30 min at 4 °C, followed by ultra-centrifugation of cytoplasmic membranes at 180,000 g for 2 h at 4 °C. Collected membranes were resuspended in membrane resuspension buffer (Supplementary Table S4) in a 1:3 (w:v) ratio prior to extraction with 2% of the soft detergent lauryl maltose neopentyl glycol (LMNG, BioNordika), at room temperature for 1 h. Extracted proteins were collected by ultra-centrifugation (180,000 g, 30 min, 4 °C), and the supernatant loaded onto a Ni-IDA column (ThermoFisher) for affinity purification, followed by size-exclusion chromatography.

### Agarose gel and SDS-PAGE

0.8% agarose gel was used to control PCR product size as well as the Gibson assembly. 500 ng of DNA were loaded together with a 6X loading dye (New England Biolabs), and run for 35 min at 120 mV. The purity of the protein samples and presence of all subunits was assessed by SDS-PAGE. 20 µg of each sample was loaded onto a mini-PROTEAN® TGX™ precast protein gel, (BioRad), and run for 30 min at constant voltage of 200 mV. Protein band were revealed by incubating the gel for 20 min in a Quick Stain solution (CliniScience).

## Supplementary Information

Supplementary Information.

## Data Availability

The sequencing results from mutagenesis experiments generated by Macrogen Europe analysed during the current study are publicly available in the following link: [https://figshare.com/s/4190e193f21cf6108b57].
