# Supplementary material for: Directed mutagenesis of large multi-subunit protein complexes by plasmid sub-fragmentation
Source: Sci Rep. 2026 May 25;16:16149. doi: 10.1038/s41598-026-53234-8 (PMC13201721; doi:10.1038/s41598-026-53234-8)
Supplement: Supplementary file 1 — Supplementary Information. [file 41598_2026_53234_MOESM1_ESM.pdf]

Supporting Information

Directed mutagenesis of large multi-subunit protein complexes by plasmid sub-fragmentation

Adel Beghiah[1], Ville R. I. Kaila[1,*]
[1] Department of Biochemistry and Biophysics, The Arrhenius Laboratories for Natural Sciences, Stockholm University, SE-106 91, Stockholm, Sweden.
* Correspondence: ville.kaila@dbb.su.se

Content

Supplementary Figures

Supplementary Fig. S1 | Uncropped agarose gel.
Supplementary Fig. S2 | pUC19 library.
Supplementary Fig. S3 | Sequencing data and multiple sequence alignment of the studied protein subunits.
Supplementary Fig. S4 | Statistical analysis of the sequencing results.
Supplementary Fig. S5 | Statistical analysis of the error rate from whole plasmid sequencing data.
Supplementary Fig. S6 | Polymerase long-range amplification efficiency for the Platinum SuperFi II (P-S), Phusion plus (P) and Q5 polymerases.
Supplementary Fig. S7 | Uncropped gels.

Supplementary Tables

Supplementary Table S1 | pBAD fragmentation protocol.
Supplementary Table S2 | Amino acid sequences of studied protein subunits from whole-plasmid sequencing data.
Supplementary Table S3 | PCR cycles parameters.
Supplementary Table S4 | PCR reaction mixtures.
Supplementary Table S5 | Composition of overexpression media and purification buffers.

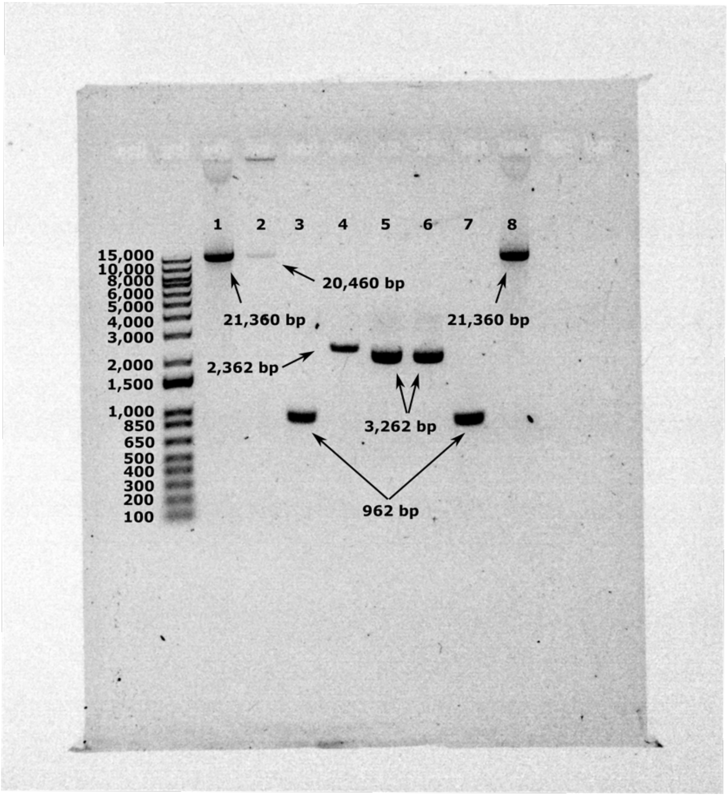

Supplementary Fig. S1 | Uncropped agarose gel showing (1) the $pBAD_{nuo}$ intact plasmid, (2) the $pBAD_{nuo}$ linearized plasmid excluding fragment 12, (3) the WT fragment 12, (4) the linearized $pUC19_{\Delta lacZ}$, (5) pUC19-12, (6) pUC19-12-E216Q^H, (7) fragment 12 of E216Q^H, and (8) the pBADnuo-E216Q^H. The circular pUC19-n is more compact and therefore migrates faster than linearised pUC19 on the gel.

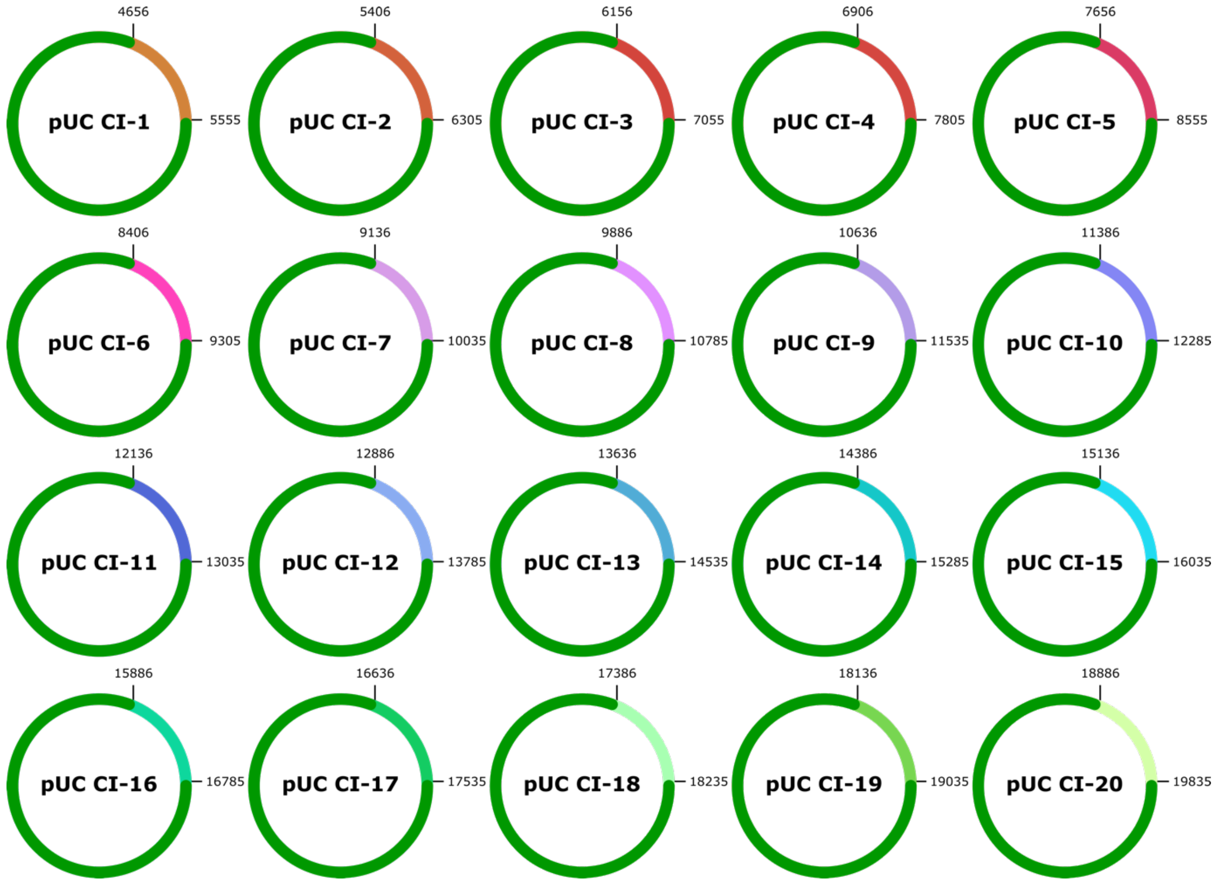

Supplementary Fig. S2 | pUC19-CI-*n* library, with the pUC19 plasmid sequence (dark green) and inserted fragment from Complex I (coloured regions). Numbers on the fragment correspond to the nucleotide positions segmented from the pBAD$_{nuo}$ plasmid.

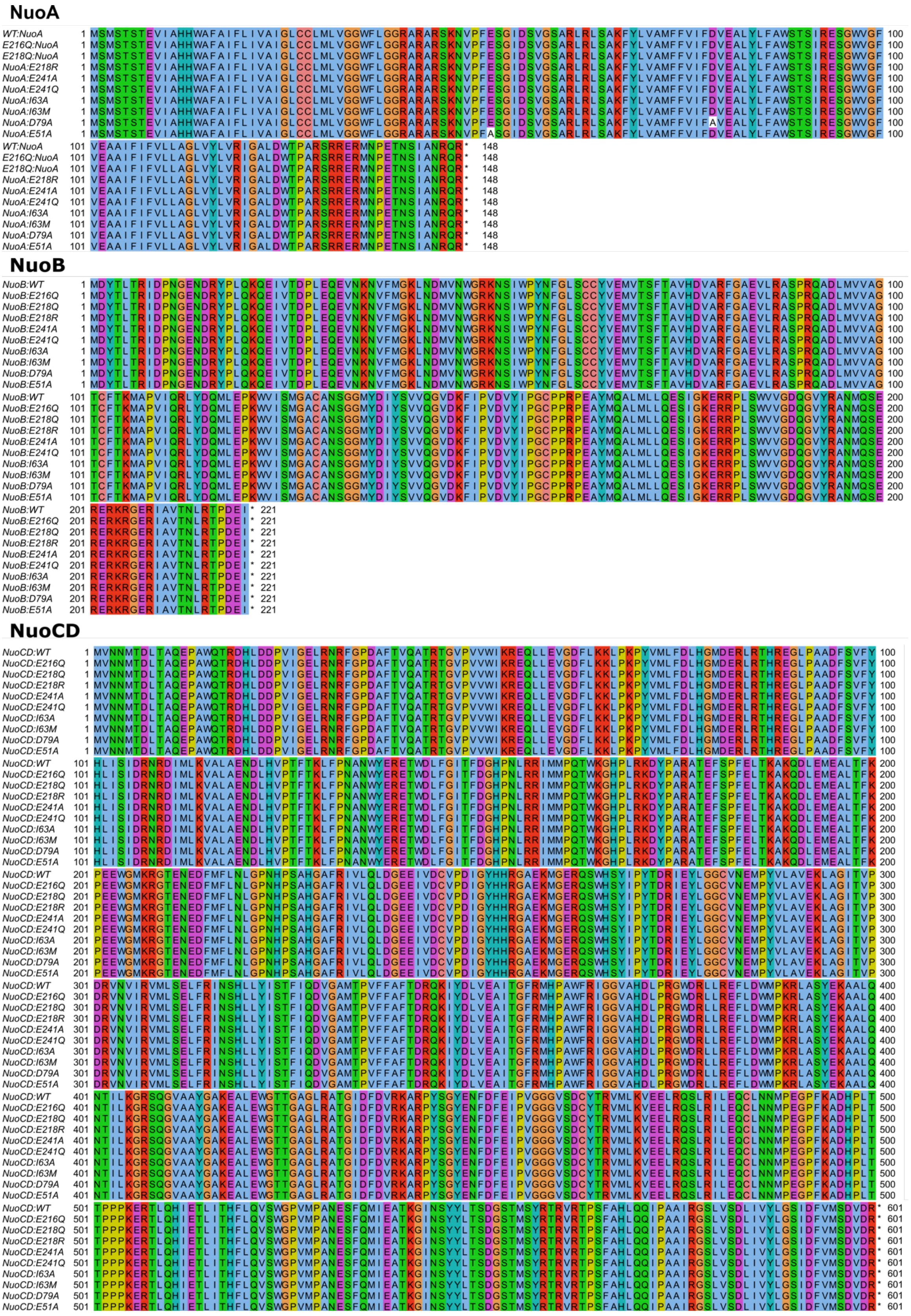

Supplementary Fig. S3 | Sequencing data and multiple sequence alignment of the DNA sequence coding for the parent enzyme (WT) and variants of *E. coli* Complex I. The alignment was performed with JalView, highlighting the sequences with the Clustal colouring scheme. The PSF approach results in a fully reproduced sequence, without unspecific mutation introduced, as shown by the whole plasmid sequencing data.

NuoE

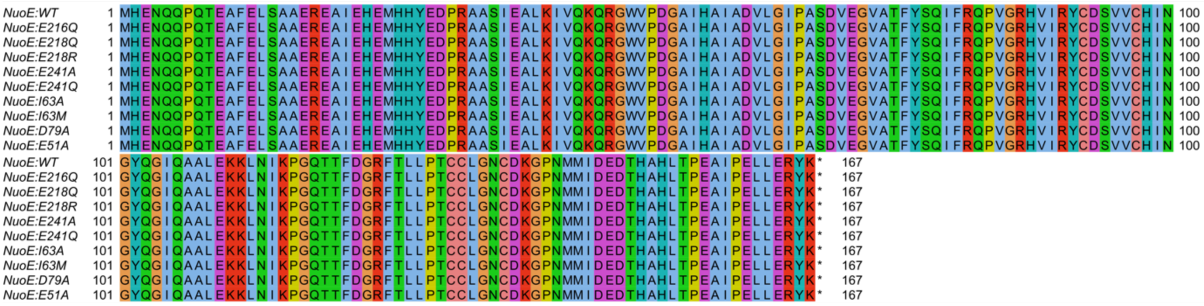

NuoF

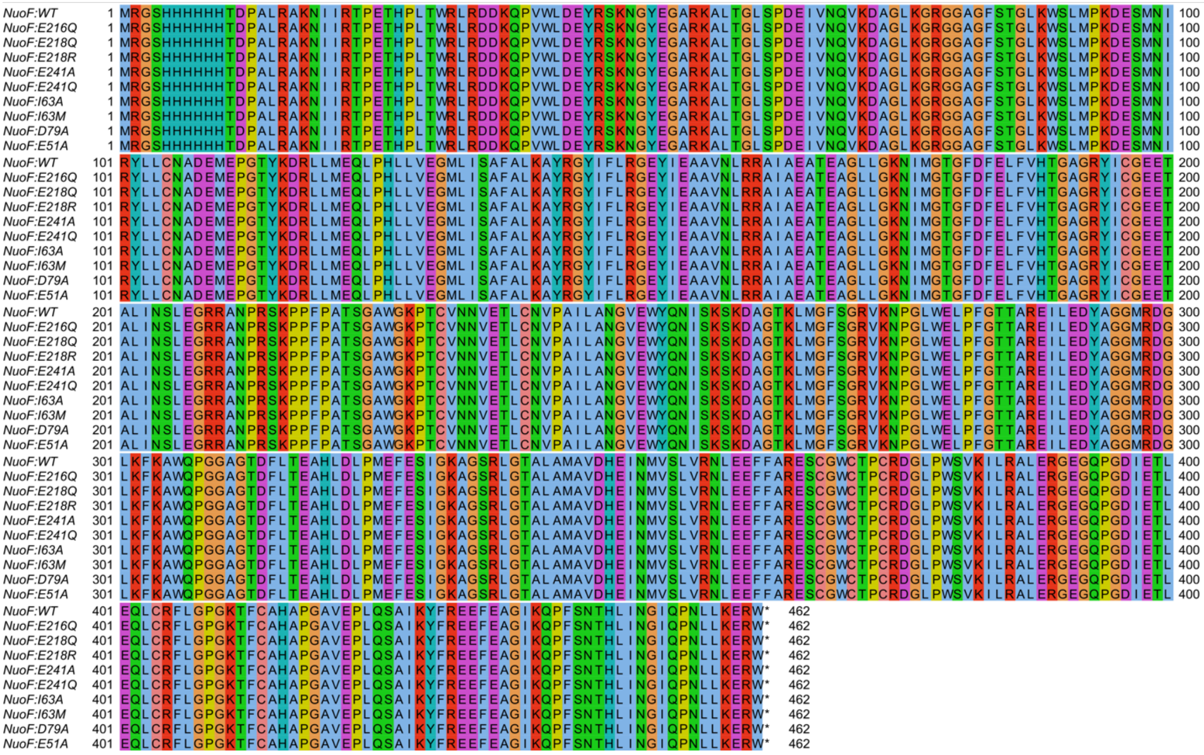

Supplementary Fig. S3 (contd) | Sequencing data and multiple sequence alignment of the DNA sequence coding for the parent enzyme (WT) and variants of *E. coli* Complex I. The alignment was performed with JalView, highlighting the sequences with the Clustal colouring scheme. The PSF approach results in a fully reproduced sequence, without unspecific mutation introduced, as shown by the whole plasmid sequencing data.

NuoG

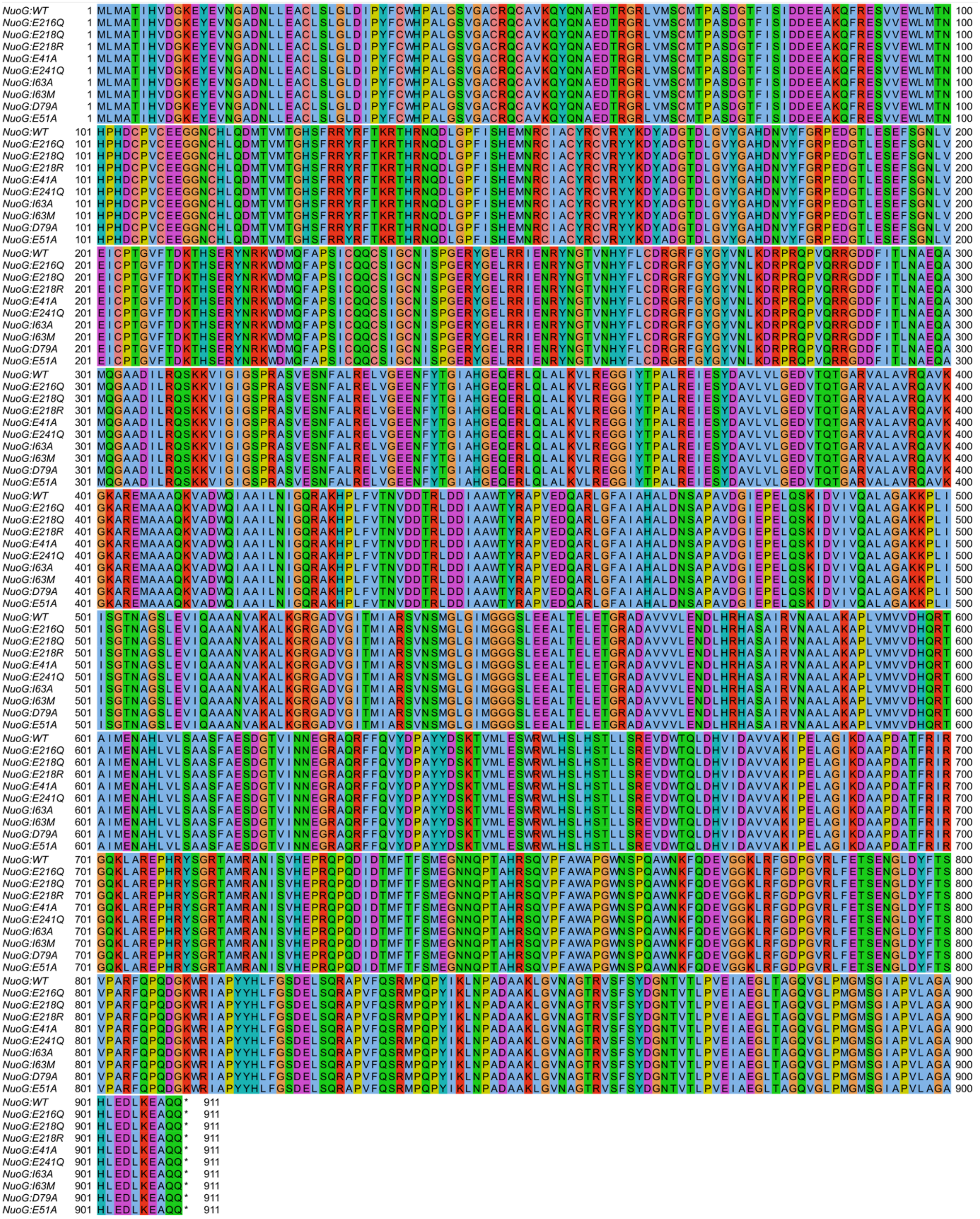

Supplementary Fig. S3 (contd.) | Sequencing data and multiple sequence alignment of the DNA sequence coding for the parent enzyme (WT) and variants of *E. coli* Complex I. The alignment was performed with JalView, highlighting the sequences with the Clustal colouring scheme. The PSF approach results in a fully reproduced sequence, without unspecific mutation introduced, as shown by the whole plasmid sequencing data.

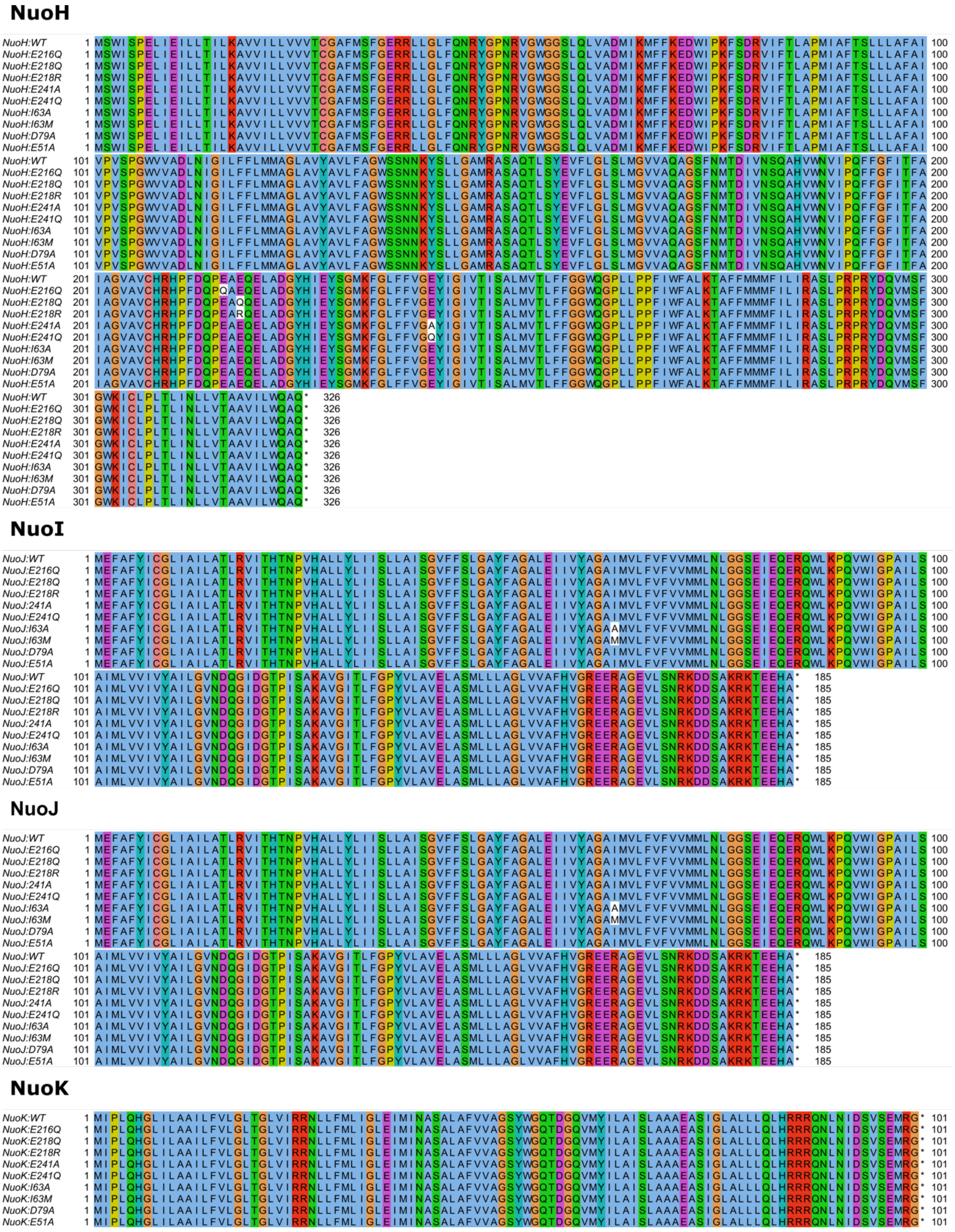

Supplementary Fig. S3 (contd.) | Sequencing data and multiple sequence alignment of the DNA sequence coding for the parent enzyme (WT) and variants of *E. coli* Complex **I**. The alignment was performed with JalView, highlighting the sequences with the Clustal colouring scheme. The PSF approach results in a fully reproduced sequence, without unspecific mutation introduced, as shown by the whole plasmid sequencing data.

NuoL

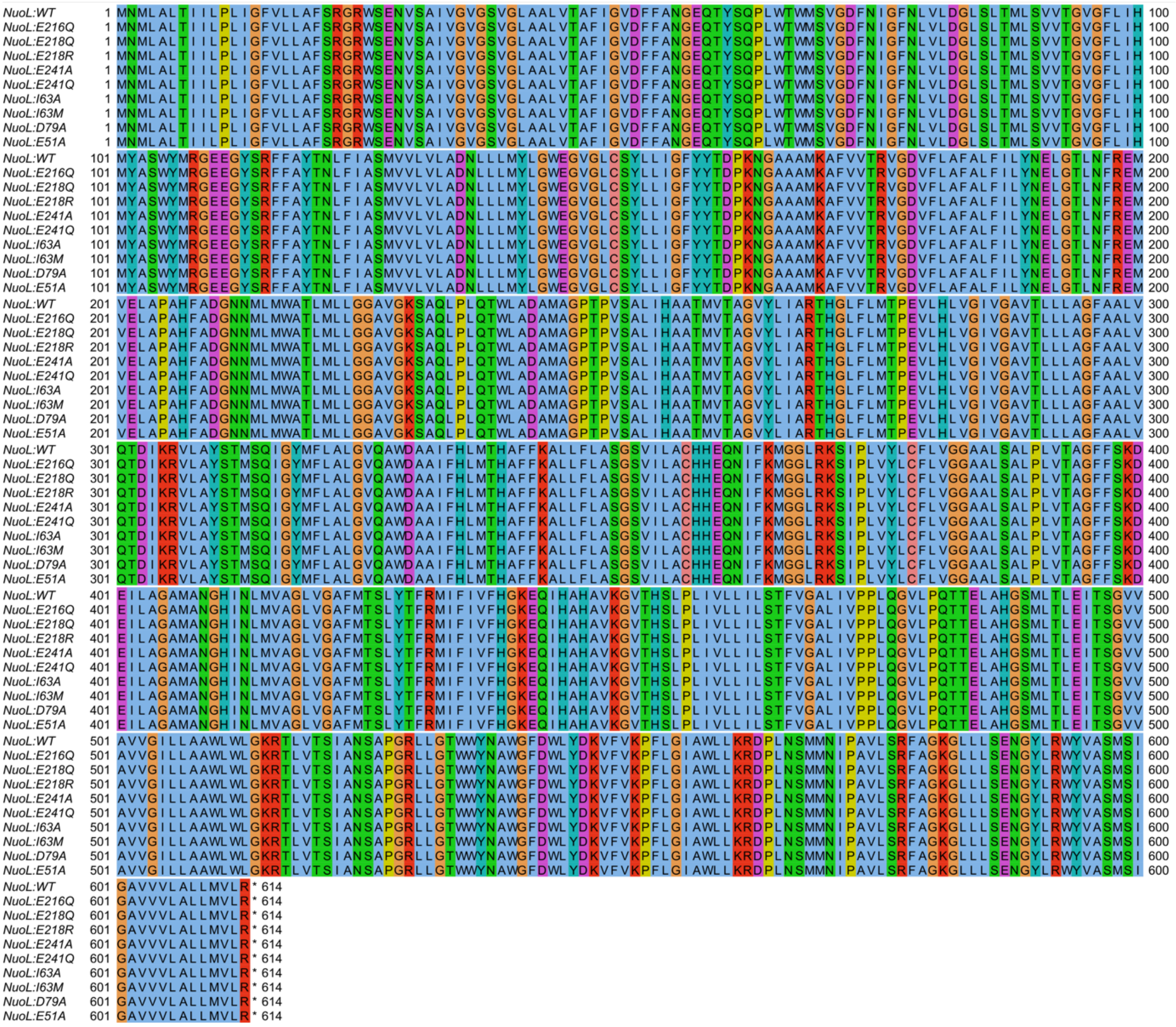

Supplementary Fig. S3 (contd.) | Sequencing data and multiple sequence alignment of the DNA sequence coding for the parent enzyme (WT) and variants of *E. coli* Complex I. The alignment was performed with JalView, highlighting the sequences with the Clustal colouring scheme. The PSF approach results in a fully reproduced sequence, without unspecific mutation introduced, as shown by the whole plasmid sequencing data.

NuoM

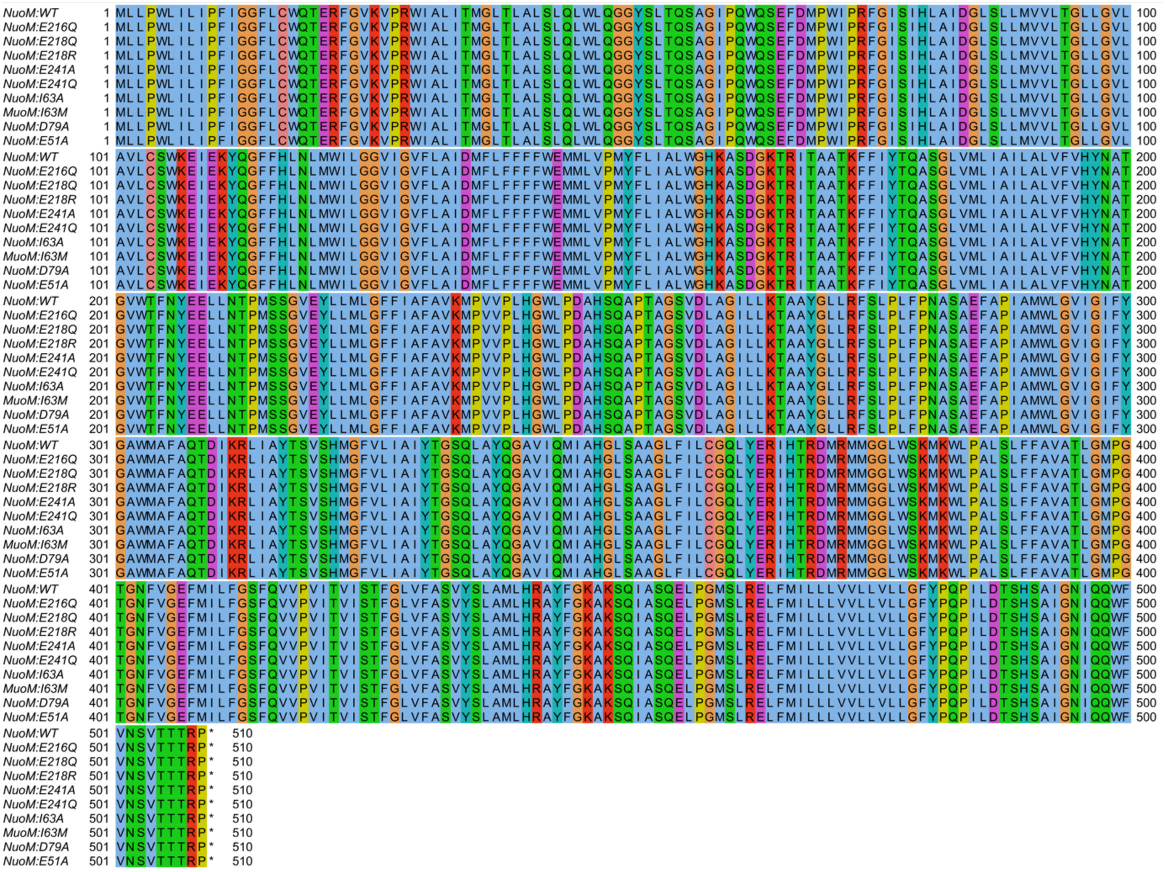

NuoN

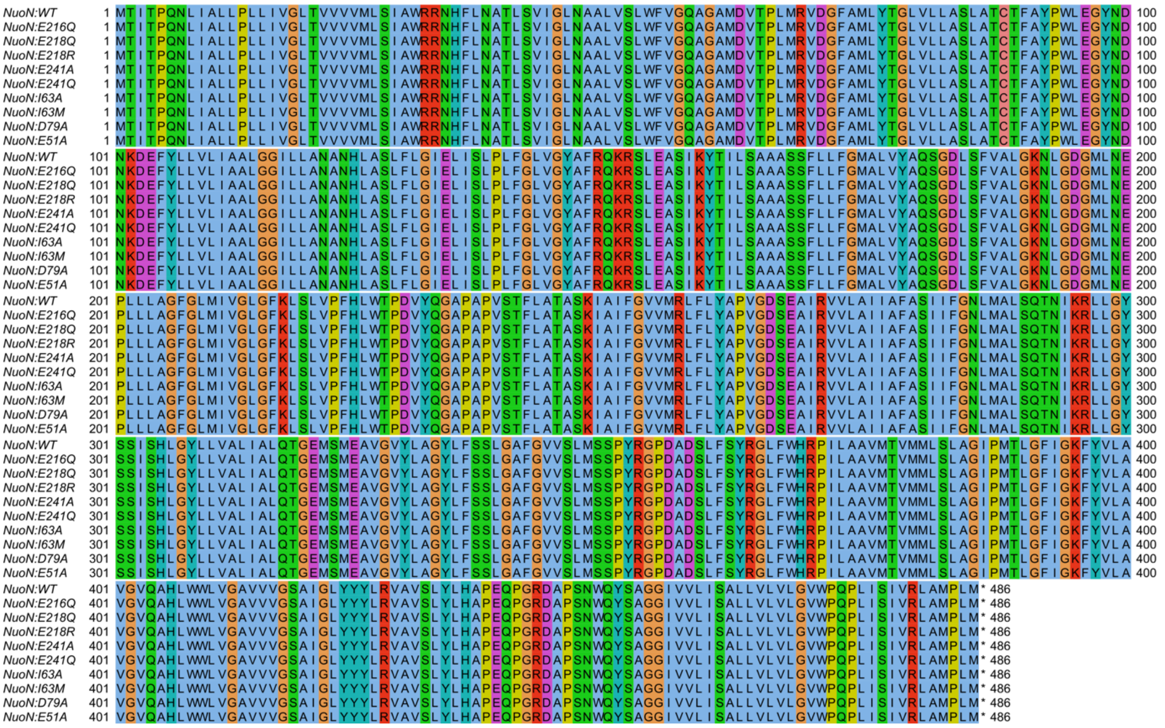

Supplementary Fig. S3 (contd.) | Sequencing data and multiple sequence alignment of the DNA sequence coding for the parent enzyme (WT) and variants of *E. coli* Complex I. The alignment was performed with JalView, highlighting the sequences with the Clustal colouring scheme. The PSF approach results in a fully reproduced sequence, without unspecific mutation introduced, as shown by the whole plasmid sequencing data

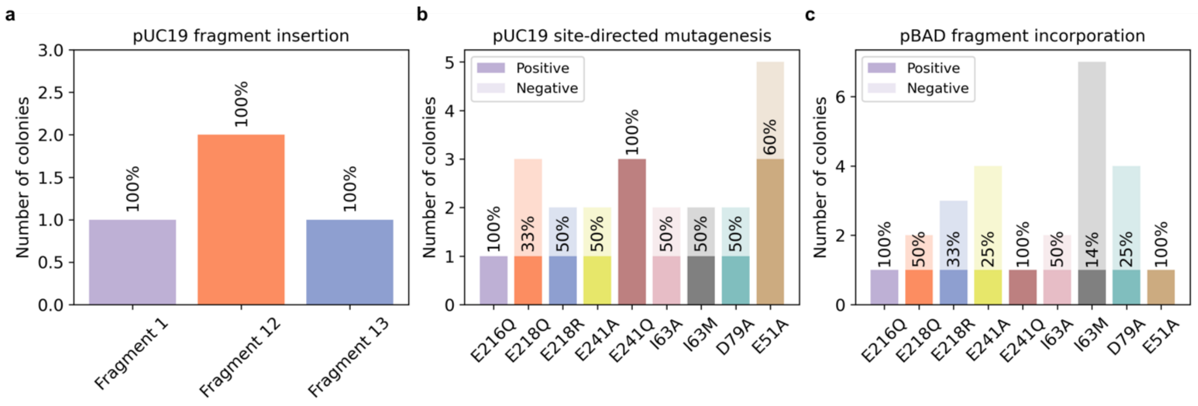

Supplementary Fig. S4 | Statistical analysis of the sequencing results. Sequencing results from a) fragment insertion into pUC19$_{\Delta LacZa}$, b) site-directed mutagenesis performed in pUC19-*n* (where *n* stands for the fragment number), and, c) incorporation of mutated fragment into the pBAD$_{nuo}$ vector.

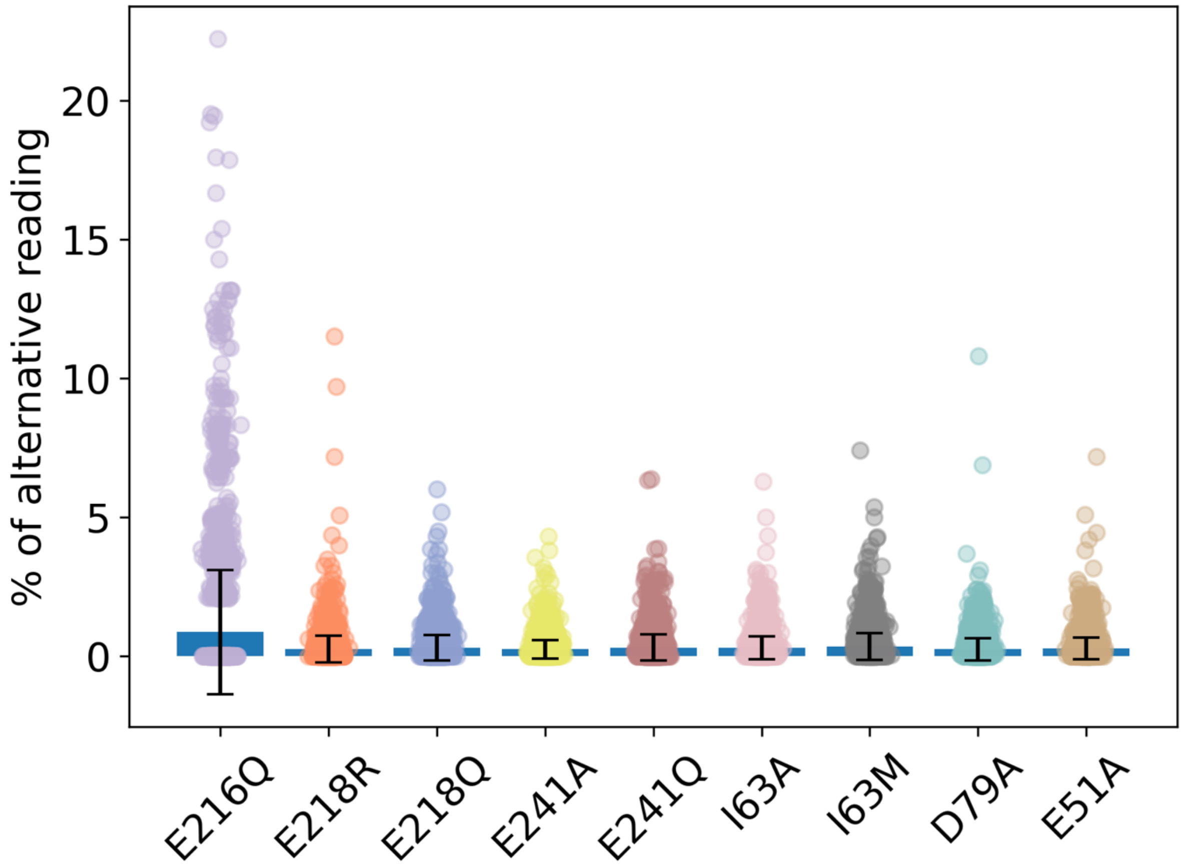

Supplementary Fig. S5 | Statistical analysis of the error rate from whole plasmid sequencing data.

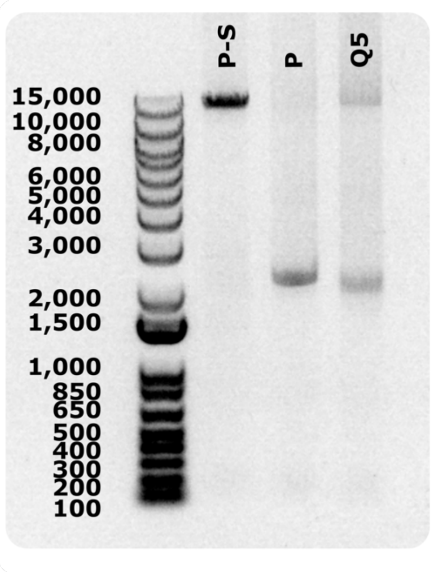

Supplementary Fig. S6 | Polymerase long-range amplification efficiency for the Platinum SuperFi II (P-S), Phusion plus (P) and Q5 polymerases.

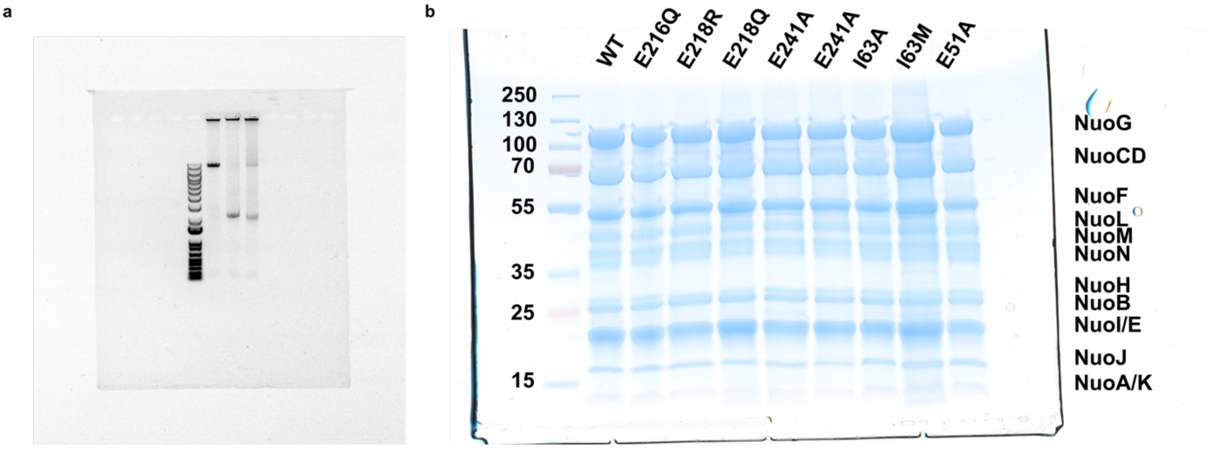

Supplementary Fig. S7 | Uncropped gels. a) Uncropped agarose gel showing PCR product from pBAD vector linearisation using the Platinum SuperFi II, Phusion plus or Q5 polymerases. b) Uncropped SDS-PAGE gel showing purified Complex I variants.

Supplementary Table S1 | pBAD fragmentation positions.

Fragment	Start position	End position	5’ overlap region	3’ overlap region
**1**	4,656	5,555	-	5,406-5,555
**2**	5,406	6,305	5,406-5,555	6,156-6,305
**3**	6,156	7,055	6,156-6,305	6,906-7,055
**4**	6,906	7,805	6,906-7,055	7,656-7,805
**5**	7,656	8,555	7,656-7,805	8,406-8,555
**6**	8,406	9,305	8,406-8,555	9,136-9,305
**7**	9,136	10,035	9,136-9,305	9,886-10,035
**8**	9,886	10,785	9,886-10,035	10,636-10,785
**9**	10,636	11,535	10,636-10,785	11,386-11,535
**10**	11,386	12,285	11,386-11,535	12,136-12,285
**11**	12,136	13,035	12,136-12,285	12,886-13,035
**12**	12,886	13,785	12,886-13,035	13,636-13,785
**13**	13,636	14,535	13,636-13,785	14,386-14,535
**14**	14,386	15,285	14,386-14,535	15,136-15,285
**15**	15,136	16,035	15,136-15,285	15,886-16,035
**16**	15,886	16,785	15,886-16,035	16,636-16,785
**17**	16,636	17,535	16,636-16,785	17,386-17,535
**18**	17,386	18,285	17,386-17,535	18,136-18,285
**19**	18,136	19,035	18,136-18,285	18,886-19,035
**20**	18,886	19,835	18,886-19,035	-

Supplementary Table S2 | Amino acid sequences of studied protein subunits from whole-plasmid sequencing data.

Complex I WT	**NuoA**<br>MSMSTSTEVIAHHWAFAIFLIVAIGLCCLMLVGGWFLGGRARARSKNVPFESGIDS<br>VGSARLRLSAKFYLVAMFFVIFDVEALYLFAWSTSIRESGWVGFVEAAIFIFVLLAG<br>LVYLVRIGALDWTPARSRRERMNPETNSIANRQR*<br><br>**NuoB**<br>MDYTLTRIDPNGENDRYPLQKQEIVTDPLEQEVNKNVFMGKLNDMVNWGRKNSI<br>WPYNFGLSCCYVEMVTSFTAVHDVARFGAEVLRASPRQADLMVVAGTCFTKMA<br>PVIQRLYDQMLEPKWVISMGACANSGGMYDIYSVVQGVDKFIPVDVYIPGCPPRP<br>EAYMQALMLLQESIGKERRPLSWVVGDQGVYRANMQSERERKRGERIAVTNLRT<br>PDEI*<br><br>**NuoCD**<br>MVNNMTDLTAQEPAWQTRDHLDDPVIGELRNRFGPDAFTVQATRTGVPVVWIKR<br>EQLLEVGDFLKKLPKPYVMLFDLHGMDERLRTHREGLPAADFSVFYHLISIDRNRD<br>IMLKVALAENDLHVPTFTKLFPNANWYERETWDLFGITFDGHPNLRRIMMPQTWK<br>GHPLRKDYPARATEFSPFELTKAKQDLEMEALTFKPEEWGMKRGTENEDFMFLNL<br>GPNHPSAHGAFRIVLQLDGEEIVDCVPDIGYHHRGAEKMGERQSWHSYIPYTDRI<br>EYLGGCVNEMPYVLAVEKLAGITVPDRVNVIRVMLSELFRINSHLLYISTFIQDVGA<br>MTPVFFAFTDRQKIYDLVEAITGFRMHPAWFRIGGVAHDLPRGWDRLLREFLDWM<br>PKRLASYEKAALQNTILKGRSQGVAAYGAKEALEWGTTGAGLRATGIDFDVRKAR<br>PYSGYENFDFEIPVGGGVSDCYTRVMLKVEELRQSLRILEQCLNNMPEGPFKADH<br>PLTTPPPKERTLQHIETLITHFLQVSWGPVMPANESFQMIEATKGINSYYLTSDGST<br>MSYRTRVRTPSFAHLQQIPAAIRGSLVSDLIVYLGSIDFVMSDVDR*<br><br>**NuoE**<br>MHENQQPQTEAFELSAAEREAIEHEMHHYEDPRAASIEALKIVQKQRGWVPDGAI<br>HAIADVLGIPASDVEGVATFYSQIFRQPVGRHVIRYCDSVVCHINGYQGIQAALEKK<br>LNIKPGQTTFDGRFTLLPTCCLGNCDKGPNMMIDEDTHAHLTPEAIPELLERYK*<br><br>**NuoF**<br>MRGSHHHHHHTDPALRAKNIIRTPETHPLTWRLRDDKQPVWLDEYRSKNGYEGA<br>RKALTGLSPDEIVNQVKDAGLKGRGGAGFSTGLKWSLMPKDESMNIRYLLCNAD<br>EMEPGTYKDRLLMEQLPHLLVEGMLISAFALKAYRGYIFLRGEYIEAAVNLRRAIAE<br>ATEAGLLGKNIMGTGFDFELFVHTGAGRYICGEETALINSLEGRRANPRSKPPFPA<br>TSGAWGKPTCVNNVETLCNVPAILANGVEWYQNISKSKDAGTKLMGFSGRVKNP<br>GLWELPFGTTAREILEDYAGGMRDGLKFKAWQPGGAGTDFLTEAHLDLPMEFESI<br>GKAGSRLGTALAMAVDHEINMVSLVRNLEEFFARESCGWCTPCRDGLPWSVKILR<br>ALERGEGQPGDIETLEQLCRFLGPGKTFCAHAPGAVEPLQSAIKYFREEFEAGIKQ<br>PFSNTHLINGIQPNLLKERW*<br><br>**NuoG**<br>MLMATIHVDGKEYEVNGADNLLEACLSLGLDIPYFCWHPALGSVGACRQCAVKQY<br>QNAEDTRGRLVMSCMTPASDGTFISIDDEEAKQFRESVVEWLMTNHPHDCPVCE<br>EGGNCHLQDMTVMTGHSFRRYRFTKRTHRNQDLGPFISHEMNRCIACYRCVRYY<br>KDYADGTDLGVYGAHDNVYFGRPEDGTLESEFSGNLVEICPTGVFTDKTHSERYN<br>RKWDMQFAPSICQQCSIGCNISPGERYGELRRIENRYNGTVNHYFLCDRGRFGY<br>GYVNLKDRPRQPVQRRGDDFITLNAEQAMQGAADILRQSKKVIGIGSPRASVESN<br>FALRELVGEENFYTGIAHGEQERLQLALKVLREGGIYTPALREIESYDAVLVLGEDV<br>TQTGARVALAVRQAVKGKAREMAAAQKVADWQIAAILNIGQRAKHPLFVTNVDDT<br>RLDDIAAWTYRAPVEDQARLGFAIAHALDNSAPAVDGIEPELQSKIDVIVQALAGAK<br>KPLIISGTNAGSLEVIQAAANVAKALKGRGADVGITMIARSVNSMGLGIMGGGSLE<br>EALTELETGRADAVVVLENDLHRHASAIRVNAALAKAPLVMVVDHQRTAIMENAHL<br>VLSAASFAESDGTVINNEGRAQRFFQVYDPAYYDSKTVMLESWRWLHSLHSTLLS<br>REVDWTQLDHVIDAVVAKIPELAGIKDAAPDATFRIRGQKLAREPHRYSGRTAMRA<br>NISVHEPRQPQDIDTMFTFSMEGNNQPTAHRSQVPFAWAPGWNSPQAWNKFQD<br>EVGGKLRFGDPGVRLFETSENGLDYFTSVPARFQPQDGKWRIAPYYHLFGSDEL

SQRAPVFQSRMPQPYIKLNPADAAKLGVNAGTRVSFSYDGNTVTLPVEIAEGLTA
GQVGLPMGMSGIAPVLAGAHLEDLKEAQQ*

NuoH
MSWISPELIEILLTILKAVVILLVVVTCGAFMSFGERRLLGLFQNRYGPNRVGWGGS
LQLVADMIKMFFKEDWIPKFSDRVIFTLAPMIAFTSLLLAFAIVPVSPGWVVADLNIGI
LFFLMMAGLAVYAVLFAGWSSNNKYSLLGAMRASAQTLSYEVFLGLSLMGVVAQA
GSFNMTDIVNSQAHVWNVIPQFFGFITFAIAGVAVCHRHPFDQPEAEQELADGYHI
EYSGMKFGLFFVGEYIGIVTISALMVTLFFGGWQGPLLPPFIWFALKTAFFMMMFIL
IRASLPRPRYDQVMSFGWKICLPLTLINLLVTAAVILWQAQ*

NuoI
MTLKELLVGFGTQVRSIWMIGLHAFAKRETRMYPEEPVYLPPRYRGRIVLTRDPD
GEERCVACNLCAVACPVGCISLQKAETKDGRWYPEFFRINFSRCIFCGLCEEACP
TTAIQLTPDFEMGEYKRQDLVYEKEDLLISGPGKYPEYNFYRMAGMAIDGKDKGE
AENEAKPIDVKSLLP*

NuoJ
MEFAFYICGLIAILATLRVITHTNPVHALLYLIISLLAISGVFFSLGAYFAGALEIIVYAGA
IMVLFVFVVMMLNLGGSEIEQERQWLKPQVWIGPAILSAIMLVVIVYAILGVNDQGI
DGTPISAKAVGITLFGPYVLAVELASMLLLAGLVVAFHVGREERAGEVLSNRKDDS
AKRKTEEHA*

NuoK
MIPLQHGLILAAILFVLGLTGLVIRRNLLFMLIGLEIMINASALAFVVAGSYWGQTDG
QVMYILAISLAAAEASIGLALLLQLHRRRQNLNIDSVSEMRG*

NuoL
MNMLALTIILPLIGFVLLAFSRGRWSENVSAIVGVGSVGLAALVTAFIGVDFFANGE
QTYSQPLWTWMSVGDFNIGFNLVLDGLSLTMLSVVTGVGFLIHMYASWYMRGEE
GYSRFFAYTNLFIASMVVLVLADNLLLMYLGWEGVGLCSYLLIGFYYTDPKNGAAA
MKAFVVTRVGDVFLAFALFILYNELGTLNFREMVELAPAHFADGNNMLMWATLMLL
GGAVGKSAQLPLQTWLADAMAGPTPVSALIHAATMVTAGVYLIARTHGLFLMTPE
VLHLVGIVGAVTLLLAGFAALVQTDIKRVLAYSTMSQIGYMFLALGVQAWDAAIFHL
MTHAFFKALLFLASGSVILACHHEQNIFKMGGLRKSIPLVYLCFLVGGAALSALPLV
TAGFFSKDEILAGAMANGHINLMVAGLVGAFMTSLYTFRMIFIVFHGKEQIHAHAVK
GVTHSLPLIVLLILSTFVGALIVPPLQGVLPQTTELAHGSMLTLEITSGVVAVVGILLA
AWLWLGKRTLVTSIANSAPGRLLGTWWYNAWGFDWLYDKVFVKPFLGIAWLLKR
DPLNSMMNIPAVLSRFAGKGLLLSENGYLRWYVASMSIGAVVVLALLMVLR*

NuoM
MLLPWLILIPFIGGFLCWQTERFGVKVPRWIALITMGLTLALSLQLWLQGGYSLTQS
AGIPQWQSEFDMPWIPRFGISIHLAIDGLSLLMVVLTGLLGVLAVLCSWKEIEKYQG
FFHLNLMWILGGVIGVFLAIDMFLFFFFWEMMLVPMYFLIALWGHKASDGKTRITAA
TKFFIYTQASGLVMLIAILALVFVHYNATGVWTFNYEELLNTPMSSGVEYLLMLGFFI
AFAVKMPVVPLHGWLPDAHSQAPTAGSVDLAGILLKTAAYGLLRFSLPLFPNASAE
FAPIAMWLGVIGIFYGAWMAFAQTDIKRLIAYTSVSHMGFVLIAIYTGSQLAYQGAVI
QMIAHGLSAAGLFILCGQLYERIHTRDMRMMGGLWSKMKWLPALSLFFAVATLGM
PGTGNFVGEFMILFGSFQVVPVITVISTFGLVFASVYSLAMLHRAYFGKAKSQIASQ
ELPGMSLRELFMILLLVVLLVLLGFYPQPILDTSHSAIGNIQQWFVNSVTTTRP*

NuoN
MTITPQNLIALLPLLIVGLTVVVVMLSIAWRRNHFLNATLSVIGLNAALVSLWFVGQA
GAMDVTPLMRVDGFAMLYTGLVLLASLATCTFAYPWLEGYNDNKDEFYLLVLIAAL
GGILLANANHLASLFLGIELISLPLFGLVGYAFRQKRSLEASIKYTILSAAASSFLLFG
MALVYAQSGDLSFVALGKNLGDGMLNEPLLLAGFGLMIVGLGFKLSLVPFHLWTP
DVYQGAPAPVSTFLATASKIAIFGVVMRLFLYAPVGDSEAIRVVLAIIAFASIIFGNLM
ALSQTNIKRLLGYSSISHLGYLLVALIALQTGEMSMEAVGVYLAGYLFSSLGAFGVV
SLMSSPYRGPDADSLFSYRGLFWHRPILAAVMTVMMLSLAGIPMTLGFIGKFYVLA
VGVQAHLWWLVGAVVVGSAIGLYYYLRVAVSLYLHAPEQPGRDAPSNWQYSAGG
IVVLISALLVLVLGVWPQPLISIVRLAMPLM*

E216Q[NuoH]	**NuoA**<br>MSMSTSTEVIAHHWAFAIFLIVAIGLCCLMLVGGWFLGGRARARSKNVPFESGIDS<br>VGSARLRLSAKFYLVAMFFVIFDVEALYLFAWSTSIRESGWVGFVEAAIFIFVLLAG<br>LVYLVRIGALDWTPARSRRERMNPETNSIANRQR*<br><br>**NuoB**<br>MDYTLTRIDPNGENDRYPLQKQEIVTDPLEQEVNKNVFMGKLNDMVNWGRKNSI<br>WPYNFGLSCCYVEMVTSFTAVHDVARFGAEVLRASPRQADLMVVAGTCFTKMA<br>PVIQRLYDQMLEPKWVISMGACANSGGMYDIYSVVQGVDKFIPVDVYIPGCPPRP<br>EAYMQALMLLQESIGKERRPLSWVVGDQGVYRANMQSERERKRGERIAVTNLRT<br>PDEI*<br><br>**NuoCD**<br>MVNNMTDLTAQEPAWQTRDHLDDPVIGELRNRFGPDAFTVQATRTGVPVVWIKR<br>EQLLEVGDFLKKLPKPYVMLFDLHGMDERLRTHREGLPAADFSVFYHLISIDRNRD<br>IMLKVALAENDLHVPTFTKLFPNANWYERETWDLFGITFDGHPNLRRIMMPQTWK<br>GHPLRKDYPARATEFSPFELTKAKQDLEMEALTFKPEEWGMKRGTENEDFMFLNL<br>GPNHPSAHGAFRIVLQLDGEEIVDCVPDIGYHHRGAEKMGERQSWHSYIPYTDRI<br>EYLGGCVNEMPYVLAVEKLAGITVPDRVNVIRVMLSELFRINSHLLYISTFIQDVGA<br>MTPVFFAFTDRQKIYDLVEAITGFRMHPAWFRIGGVAHDLPRGWDRLLREFLDWM<br>PKRLASYEKAALQNTILKGRSQGVAAYGAKEALEWGTTGAGLRATGIDFDVRKAR<br>PYSGYENFDFEIPVGGGVSDCYTRVMLKVEELRQSLRILEQCLNNMPEGPFKADH<br>PLTTPPPKERTLQHIETLITHFLQVSWGPVMPANESFQMIEATKGINSYYLTSDGST<br>MSYRTRVRTPSFAHLQQIPAAIRGSLVSDLIVYLGSIDFVMSDVDR*<br><br>**NuoE**<br>MHENQQPQTEAFELSAAEREAIEHEMHHYEDPRAASIEALKIVQKQRGWVPDGAI<br>HAIADVLGIPASDVEGVATFYSQIFRQPVGRHVIRYCDSVVCHINGYQGIQAALEKK<br>LNIKPGQTTFDGRFTLLPTCCLGNCDKGPNMMIDEDTHAHLTPEAIPELLERYK*<br><br>**NuoF**<br>MRGSHHHHHHTDPALRAKNIIRTPETHPLTWRLRDDKQPVWLDEYRSKNGYEGA<br>RKALTGLSPDEIVNQVKDAGLKGRGGAGFSTGLKWSLMPKDESMNIRYLLCNAD<br>EMEPGTYKDRLLMEQLPHLLVEGMLISAFALKAYRGYIFLRGEYIEAAVNLRRAIAE<br>ATEAGLLGKNIMGTGFDFELFVHTGAGRYICGEETALINSLEGRRANPRSKPPFPA<br>TSGAWGKPTCVNNVETLCNVPAILANGVEWYQNISKSKDAGTKLMGFSGRVKNP<br>GLWELPFGTTAREILEDYAGGMRDGLKFKAWQPGGAGTDFLTEAHLDLPMEFESI<br>GKAGSRLGTALAMAVDHEINMVSLVRNLEEFFARESCGWCTPCRDGLPWSVKILR<br>ALERGEGQPGDIETLEQLCRFLGPGKTFCAHAPGAVEPLQSAIKYFREEFEAGIKQ<br>PFSNTHLINGIQPNLLKERW*<br><br>**NuoG**<br>MLMATIHVDGKEYEVNGADNLLEACLSLGLDIPYFCWHPALGSVGACRQCAVKQY<br>QNAEDTRGRLVMSCMTPASDGTFISIDDEEAKQFRESVVEWLMTNHPHDCPVCE<br>EGGNCHLQDMTVMTGHSFRRYRFTKRTHRNQDLGPFISHEMNRCIACYRCVRYY<br>KDYADGTDLGVYGAHDNVYFGRPEDGTLESEFSGNLVEICPTGVFTDKTHSERYN<br>RKWDMQFAPSICQQCSIGCNISPGERYGELRRIENRYNGTVNHYFLCDRGRFGY<br>GYVNLKDRPRQPVQRRGDDFITLNAEQAMQGAADILRQSKKVIGIGSPRASVESN<br>FALRELVGEENFYTGIAHGEQERLQLALKVLREGGIYTPALREIESYDAVLVLGEDV<br>TQTGARVALAVRQAVKGKAREMAAAQKVADWQIAAILNIGQRAKHPLFVTNVDDT<br>RLDDIAAWTYRAPVEDQARLGFAIAHALDNSAPAVDGIEPELQSKIDVIVQALAGAK<br>KPLIISGTNAGSLEVIQAAANVAKALKGRGADVGITMIARSVNSMGLGIMGGGSLE<br>EALTELETGRADAVVVLENDLHRHASAIRVNAALAKAPLVMVVDHQRTAIMENAHL<br>VLSAASFAESDGTVINNEGRAQRFFQVYDPAYYDSKTVMLESWRWLHSLHSTLLS<br>REVDWTQLDHVIDAVVAKIPELAGIKDAAPDATFRIRGQKLAREPHRYSGRTAMRA<br>NISVHEPRQPQDIDTMFTFSMEGNNQPTAHRSQVPFAWAPGWNSPQAWNKFQD<br>EVGGKLRFGDPGVRLFETSENGLDYFTSVPARFQPQDGKWRIAPYYHLFGSDEL

SQRAPVFQSRMPQPYIKLNPADAAKLGVNAGTRVSFSYDGNTVTLPVEIAEGLTA
GQVGLPMGMSGIAPVLAGAHLEDLKEAQQ*

NuoH
MSWISPELIEILLTILKAVVILLVVVTCGAFMSFGERRLLGLFQNRYGPNRVGWGGS
LQLVADMIKMFFKEDWIPKFSDRVIFTLAPMIAFTSLLLAFAIVPVSPGWVVADLNIGI
LFFLMMAGLAVYAVLFAGWSSNNKYSLLGAMRASAQTLSYEVFLGLSLMGVVAQA
GSFNMTDIVNSQAHVWNVIPQFFGFITFAIAGVAVCHRHPFDQPQAEQELADGYHI
EYSGMKFGLFFVGEYIGIVTISALMVTLFFGGWQGPLLPPFIWFALKTAFFMMMFIL
IRASLPRPRYDQVMSFGWKICLPLTLINLLVTAAVILWQAQ*

NuoI
MTLKELLVGFGTQVRSIWMIGLHAFAKRETRMYPEEPVYLPPRYRGRIVLTRDPD
GEERCVACNLCAVACPVGCISLQKAETKDGRWYPEFFRINFSRCIFCGLCEEACP
TTAIQLTPDFEMGEYKRQDLVYEKEDLLISGPGKYPEYNFYRMAGMAIDGKDKGE
AENEAKPIDVKSLLP*

NuoJ
MEFAFYICGLIAILATLRVITHTNPVHALLYLIISLLAISGVFFSLGAYFAGALEIIVYAGA
IMVLFVFVVMMLNLGGSEIEQERQWLKPQVWIGPAILSAIMLVVIVYAILGVNDQGI
DGTPISAKAVGITLFGPYVLAVELASMLLLAGLVVAFHVGREERAGEVLSNRKDDS
AKRKTEEHA*

NuoK
MIPLQHGLILAAILFVLGLTGLVIRRNLLFMLIGLEIMINASALAFVVAGSYWGQTDG
QVMYILAISLAAAEASIGLALLLQLHRRRQNLNIDSVSEMRG*

NuoL
MNMLALTIILPLIGFVLLAFSRGRWSENVSAIVGVGSVGLAALVTAFIGVDFFANGE
QTYSQPLWTWMSVGDFNIGFNLVLDGLSLTMLSVVTGVGFLIHMYASWYMRGEE
GYSRFFAYTNLFIASMVVLVLADNLLLMYLGWEGVGLCSYLLIGFYYTDPKNGAAA
MKAFVVTRVGDVFLAFALFILYNELGTLNFREMVELAPAHFADGNNMLMWATLMLL
GGAVGKSAQLPLQTWLADAMAGPTPVSALIHAATMVTAGVYLIARTHGLFLMTPE
VLHLVGIVGAVTLLLAGFAALVQTDIKRVLAYSTMSQIGYMFLALGVQAWDAAIFHL
MTHAFFKALLFLASGSVILACHHEQNIFKMGGLRKSIPLVYLCFLVGGAALSALPLV
TAGFFSKDEILAGAMANGHINLMVAGLVGAFMTSLYTFRMIFIVFHGKEQIHAHAVK
GVTHSLPLIVLLILSTFVGALIVPPLQGVLPQTTELAHGSMLTLEITSGVVAVVGILLA
AWLWLGKRTLVTSIANSAPGRLLGTWWYNAWGFDWLYDKVFVKPFLGIAWLLKR
DPLNSMMNIPAVLSRFAGKGLLLSENGYLRWYVASMSIGAVVVLALLMVLR*

NuoM
MLLPWLILIPFIGGFLCWQTERFGVKVPRWIALITMGLTLALSLQLWLQGGYSLTQS
AGIPQWQSEFDMPWIPRFGISIHLAIDGLSLLMVVLTGLLGVLAVLCSWKEIEKYQG
FFHLNLMWILGGVIGVFLAIDMFLFFFFWEMMLVPMYFLIALWGHKASDGKTRITAA
TKFFIYTQASGLVMLIAILALVFVHYNATGVWTFNYEELLNTPMSSGVEYLLMLGFFI
AFAVKMPVVPLHGWLPDAHSQAPTAGSVDLAGILLKTAAYGLLRFSLPLFPNASAE
FAPIAMWLGVIGIFYGAWMAFAQTDIKRLIAYTSVSHMGFVLIAIYTGSQLAYQGAVI
QMIAHGLSAAGLFILCGQLYERIHTRDMRMMGGLWSKMKWLPALSLFFAVATLGM
PGTGNFVGEFMILFGSFQVVPVITVISTFGLVFASVYSLAMLHRAYFGKAKSQIASQ
ELPGMSLRELFMILLLVVLLVLLGFYPQPILDTSHSAIGNIQQWFVNSVTTTRP*

NuoN
MTITPQNLIALLPLLIVGLTVVVVMLSIAWRRNHFLNATLSVIGLNAALVSLWFVGQA
GAMDVTPLMRVDGFAMLYTGLVLLASLATCTFAYPWLEGYNDNKDEFYLLVLIAAL
GGILLANANHLASLFLGIELISLPLFGLVGYAFRQKRSLEASIKYTILSAAASSFLLFG
MALVYAQSGDLSFVALGKNLGDGMLNEPLLLAGFGLMIVGLGFKLSLVPFHLWTP
DVYQGAPAPVSTFLATASKIAIFGVVMRLFLYAPVGDSEAIRVVLAIIAFASIIFGNLM
ALSQTNIKRLLGYSSISHLGYLLVALIALQTGEMSMEAVGVYLAGYLFSSLGAFGVV
SLMSSPYRGPDADSLFSYRGLFWHRPILAAVMTVMMLSLAGIPMTLGFIGKFYVLA

	VGVQAHLWWLVGAVVVGSAIGLYYYLRVAVSLYLHAPEQPGRDAPSNWQYSAGG<br>IVVLISALLVLVLGVWPQPLISIVRLAMPLM*
E218Q^{NuoH}	**NuoA**<br>MSMSTSTEVIAHHWAFAIFLIVAIGLCCLMLVGGWFLGGRARARSKNVPFESGIDS<br>VGSARLRLSAKFYLVAMFFVIFDVEALYLFAWSTSIRESGWVGFVEAAIFIFVLLAG<br>LVYLVRIGALDWTPARSRRERMNPETNSIANRQR*<br><br>**NuoB**<br>MDYTLTRIDPNGENDRYPLQKQEIVTDPLEQEVNKNVFMGKLNDMVNWGRKNSI<br>WPYNFGLSCCYVEMVTSFTAVHDVARFGAEVLRASPRQADLMVVAGTCFTKMA<br>PVIQRLYDQMLEPKWVISMGACANSGGMYDIYSVVQGVDKFIPVDVYIPGCPPRP<br>EAYMQALMLLQESIGKERRPLSWVVGDQGVYRANMQSERERKRGERIAVTNLRT<br>PDEI*<br><br>**NuoCD**<br>MVNNMTDLTAQEPAWQTRDHLDDPVIGELRNRFGPDAFTVQATRTGVPVVWIKR<br>EQLLEVGDFLKKLPKPYVMLFDLHGMDERLRTHREGLPAADFSVFYHLISIDRNRD<br>IMLKVALAENDLHVPTFTKLFPNANWYERETWDLFGITFDGHPNLRRIMMPQTWK<br>GHPLRKDYPARATEFSPFELTKAKQDLEMEALTFKPEEWGMKRGTENEDFMFLNL<br>GPNHPSAHGAFRIVLQLDGEEIVDCVPDIGYHHRGAEKMGERQSWHSYIPYTDRI<br>EYLGGCVNEMPYVLAVEKLAGITVPDRVNVIRVMLSELFRINSHLLYISTFIQDVGA<br>MTPVFFAFTDRQKIYDLVEAITGFRMHPAWFRIGGVAHDLPRGWDRLLREFLDWM<br>PKRLASYEKAALQNTILKGRSQGVAAYGAKEALEWGTTGAGLRATGIDFDVRKAR<br>PYSGYENFDFEIPVGGGVSDCYTRVMLKVEELRQSLRILEQCLNNMPEGPFKADH<br>PLTTPPPKERTLQHIETLITHFLQVSWGPVMPANESFQMIEATKGINSYYLTSDGST<br>MSYRTRVRTPSFAHLQQIPAAIRGSLVSDLIVYLGSIDFVMSDVDR*<br><br>**NuoE**<br>MHENQQPQTEAFELSAAEREAIEHEMHHYEDPRAASIEALKIVQKQRGWVPDGAI<br>HAIADVLGIPASDVEGVATFYSQIFRQPVGRHVIRYCDSVVCHINGYQGIQAALEKK<br>LNIKPGQTTFDGRFTLLPTCCLGNCDKGPNMMIDEDTHAHLTPEAIPELLERYK*<br><br>**NuoF**<br>MRGSHHHHHHTDPALRAKNIIRTPETHPLTWRLRDDKQPVWLDEYRSKNGYEGA<br>RKALTGLSPDEIVNQVKDAGLKGRGGAGFSTGLKWSLMPKDESMNIRYLLCNAD<br>EMEPGTYKDRLLMEQLPHLLVEGMLISAFALKAYRGYIFLRGEYIEAAVNLRRAIAE<br>ATEAGLLGKNIMGTGFDFELFVHTGAGRYICGEETALINSLEGRRANPRSKPPFPA<br>TSGAWGKPTCVNNVETLCNVPAILANGVEWYQNISKSKDAGTKLMGFSGRVKNP<br>GLWELPFGTTAREILEDYAGGMRDGLKFKAWQPGGAGTDFLTEAHLDLPMEFESI<br>GKAGSRLGTALAMAVDHEINMVSLVRNLEEFFARESCGWCTPCRDGLPWSVKILR<br>ALERGEGQPGDIETLEQLCRFLGPGKTFCAHAPGAVEPLQSAIKYFREEFEAGIKQ<br>PFSNTHLINGIQPNLLKERW*<br><br>**NuoG**<br>MLMATIHVDGKEYEVNGADNLLEACLSLGLDIPYFCWHPALGSVGACRQCAVKQY<br>QNAEDTRGRLVMSCMTPASDGTFISIDDEEAKQFRESVVEWLMTNHPHDCPVCE<br>EGGNCHLQDMTVMTGHSFRRYRFTKRTHRNQDLGPFISHEMNRCIACYRCVRYY<br>KDYADGTDLGVYGAHDNVYFGRPEDGTLESEFSGNLVEICPTGVFTDKTHSERYN<br>RKWDMQFAPSICQQCSIGCNISPGERYGELRRIENRYNGTVNHYFLCDRGRFGY<br>GYVNLKDRPRQPVQRRGDDFITLNAEQAMQGAADILRQSKKVIGIGSPRASVESN<br>FALRELVGEENFYTGIAHGEQERLQLALKVLREGGIYTPALREIESYDAVLVLGEDV<br>TQTGARVALAVRQAVKGKAREMAAAQKVADWQIAAILNIGQRAKHPLFVTNVDDT<br>RLDDIAAWTYRAPVEDQARLGFAIAHALDNSAPAVDGIEPELQSKIDVIVQALAGAK<br>KPLIISGTNAGSLEVIQAAANVAKALKGRGADVGITMIARSVNSMGLGIMGGGSLE<br>EALTELETGRADAVVVLENDLHRHASAIRVNAALAKAPLVMVVDHQRTAIMENAHL<br>VLSAASFAESDGTVINNEGRAQRFFQVYDPAYYDSKTVMLESWRWLHSLHSTLLS<br>REVDWTQLDHVIDAVVAKIPELAGIKDAAPDATFRIRGQKLAREPHRYSGRTAMRA<br>NISVHEPRQPQDIDTMFTFSMEGNNQPTAHRSQVPFAWAPGWNSPQAWNKFQD

EVGGKLRFGDPGVRLFETSENGLDYFTSVPARFQPQDGKWRIAPYYHLFGSDEL
SQRAPVFQSRMPQPYIKLNPADAAKLGVNAGTRVSFSYDGNTVTLPVEIAEGLTA
GQVGLPMGMSGIAPVLAGAHLEDLKEAQQ*

NuoH
MSWISPELIEILLTILKAVVILLVVVTCGAFMSFGERRLLGLFQNRYGPNRVGWGGS
LQLVADMIKMFFKEDWIPKFSDRVIFTLAPMIAFTSLLLAFAIVPVSPGWVVADLNIGI
LFFLMMAGLAVYAVLFAGWSSNNKYSLLGAMRASAQTLSYEVFLGLSLMGVVAQA
GSFNMTDIVNSQAHVWNVIPQFFGFITFAIAGVAVCHRHPFDQPEAQQELADGYHI
EYSGMKFGLFFVGEYIGIVTISALMVTLFFGGWQGPLLPPFIWFALKTAFFMMMFIL
IRASLPRPRYDQVMSFGWKICLPLTLINLLVTAAVILWQAQ*

NuoI
MTLKELLVGFGTQVRSIWMIGLHAFAKRETRMYPEEPVYLPPRYRGRIVLTRDPD
GEERCVACNLCAVACPVGCISLQKAETKDGRWYPEFFRINFSRCIFCGLCEEACP
TTAIQLTPDFEMGEYKRQDLVYEKEDLLISGPGKYPEYNFYRMAGMAIDGKDKGE
AENEAKPIDVKSLLP*

NuoJ
MEFAFYICGLIAILATLRVITHTNPVHALLYLIISLLAISGVFFSLGAYFAGALEIIVYAGA
IMVLFVFVVMMLNLGGSEIEQERQWLKPQVWIGPAILSAIMLVVIVYAILGVNDQGI
DGTPISAKAVGITLFGPYVLAVELASMLLLAGLVVAFHVGREERAGEVLSNRKDDS
AKRKTEEHA*

NuoK
MIPLQHGLILAAILFVLGLTGLVIRRNLLFMLIGLEIMINASALAFVVAGSYWGQTDG
QVMYILAISLAAAEASIGLALLLQLHRRRQNLNIDSVSEMRG*

NuoL
MNMLALTIILPLIGFVLLAFSRGRWSENVSAIVGVGSVGLAALVTAFIGVDFFANGE
QTYSQPLWTWMSVGDFNIGFNLVLDGLSLTMLSVVTGVGFLIHMYASWYMRGEE
GYSRFFAYTNLFIASMVVLVLADNLLLMYLGWEGVGLCSYLLIGFYYTDPKNGAAA
MKAFVVTRVGDVFLAFALFILYNELGTLNFREMVELAPAHFADGNNMLMWATLMLL
GGAVGKSAQLPLQTWLADAMAGPTPVSALIHAATMVTAGVYLIARTHGLFLMTPE
VLHLVGIVGAVTLLLAGFAALVQTDIKRVLAYSTMSQIGYMFLALGVQAWDAAIFHL
MTHAFFKALLFLASGSVILACHHEQNIFKMGGLRKSIPLVYLCFLVGGAALSALPLV
TAGFFSKDEILAGAMANGHINLMVAGLVGAFMTSLYTFRMIFIVFHGKEQIHAHAVK
GVTHSLPLIVLLILSTFVGALIVPPLQGVLPQTTELAHGSMLTLEITSGVVAVVGILLA
AWLWLGKRTLVTSIANSAPGRLLGTWWYNAWGFDWLYDKVFVKPFLGIAWLLKR
DPLNSMMNIPAVLSRFAGKGLLLSENGYLRWYVASMSIGAVVVLALLMVLR*

NuoM
MLLPWLILIPFIGGFLCWQTERFGVKVPRWIALITMGLTLALSLQLWLQGGYSLTQS
AGIPQWQSEFDMPWIPRFGISIHLAIDGLSLLMVVLTGLLGVLAVLCSWKEIEKYQG
FFHLNLMWILGGVIGVFLAIDMFLFFFFWEMMLVPMYFLIALWGHKASDGKTRITAA
TKFFIYTQASGLVMLIAILALVFVHYNATGVWTFNYEELLNTPMSSGVEYLLMLGFFI
AFAVKMPVVPLHGWLPDAHSQAPTAGSVDLAGILLKTAAYGLLRFSLPLFPNASAE
FAPIAMWLGVIGIFYGAWMAFAQTDIKRLIAYTSVSHMGFVLIAIYTGSQLAYQGAVI
QMIAHGLSAAGLFILCGQLYERIHTRDMRMMGGLWSKMKWLPALSLFFAVATLGM
PGTGNFVGEFMILFGSFQVVPVITVISTFGLVFASVYSLAMLHRAYFGKAKSQIASQ
ELPGMSLRELFMILLLVVLLVLLGFYPQPILDTSHSAIGNIQQWFVNSVTTTRP*

NuoN
MTITPQNLIALLPLLIVGLTVVVVMLSIAWRRNHFLNATLSVIGLNAALVSLWFVGQA
GAMDVTPLMRVDGFAMLYTGLVLLASLATCTFAYPWLEGYNDNKDEFYLLVLIAAL
GGILLANANHLASLFLGIELISLPLFGLVGYAFRQKRSLEASIKYTILSAAASSFLLFG
MALVYAQSGDLSFVALGKNLGDGMLNEPLLLAGFGLMIVGLGFKLSLVPFHLWTP
DVYQGAPAPVSTFLATASKIAIFGVVMRLFLYAPVGDSEAIRVVLAIIAFASIIFGNLM
ALSQTNIKRLLGYSSISHLGYLLVALIALQTGEMSMEAVGVYLAGYLFSSLGAFGVV
SLMSSPYRGPDADSLFSYRGLFWHRPILAAVMTVMMLSLAGIPMTLGFIGKFYVLA

	VGVQAHLWWLVGAVVVGSAIGLYYYLRVAVSLYLHAPEQPGRDAPSNWQYSAGG<br>IVVLISALLVLVLGVWPQPLISIVRLAMPLM*
E218R^{NuoH}	**NuoA**<br>MSMSTSTEVIAHHWAFAIFLIVAIGLCCLMLVGGWFLGGRARARSKNVPFESGIDS<br>VGSARLRLSAKFYLVAMFFVIFDVEALYLFAWSTSIRESGWVGFVEAAIFIFVLLAG<br>LVYLVRIGALDWTPARSRRERMNPETNSIANRQR*<br><br>**NuoB**<br>MDYTLTRIDPNGENDRYPLQKQEIVTDPLEQEVNKNVFMGKLNDMVNWGRKNSI<br>WPYNFGLSCCYVEMVTSFTAVHDVARFGAEVLRASPRQADLMVVAGTCFTKMA<br>PVIQRLYDQMLEPKWVISMGACANSGGMYDIYSVVQGVDKFIPVDVYIPGCPPRP<br>EAYMQALMLLQESIGKERRPLSWVVGDQGVYRANMQSERERKRGERIAVTNLRT<br>PDEI*<br><br>**NuoCD**<br>MVNNMTDLTAQEPAWQTRDHLDDPVIGELRNRFGPDAFTVQATRTGVPVVWIKR<br>EQLLEVGDFLKKLPKPYVMLFDLHGMDERLRTHREGLPAADFSVFYHLISIDRNRD<br>IMLKVALAENDLHVPTFTKLFPNANWYERETWDLFGITFDGHPNLRRIMMPQTWK<br>GHPLRKDYPARATEFSPFELTKAKQDLEMEALTFKPEEWGMKRGTENEDFMFLNL<br>GPNHPSAHGAFRIVLQLDGEEIVDCVPDIGYHHRGAEKMGERQSWHSYIPYTDRI<br>EYLGGCVNEMPYVLAVEKLAGITVPDRVNVIRVMLSELFRINSHLLYISTFIQDVGA<br>MTPVFFAFTDRQKIYDLVEAITGFRMHPAWFRIGGVAHDLPRGWDRLLREFLDWM<br>PKRLASYEKAALQNTILKGRSQGVAAYGAKEALEWGTTGAGLRATGIDFDVRKAR<br>PYSGYENFDFEIPVGGGVSDCYTRVMLKVEELRQSLRILEQCLNNMPEGPFKADH<br>PLTTPPPKERTLQHIETLITHFLQVSWGPVMPANESFQMIEATKGINSYYLTSDGST<br>MSYRTRVRTPSFAHLQQIPAAIRGSLVSDLIVYLGSIDFVMSDVDR*<br><br>**NuoE**<br>MHENQQPQTEAFELSAAEREAIEHEMHHYEDPRAASIEALKIVQKQRGWVPDGAI<br>HAIADVLGIPASDVEGVATFYSQIFRQPVGRHVIRYCDSVVCHINGYQGIQAALEKK<br>LNIKPGQTTFDGRFTLLPTCCLGNCDKGPNMMIDEDTHAHLTPEAIPELLERYK*<br><br>**NuoF**<br>MRGSHHHHHHTDPALRAKNIIRTPETHPLTWRLRDDKQPVWLDEYRSKNGYEGA<br>RKALTGLSPDEIVNQVKDAGLKGRGGAGFSTGLKWSLMPKDESMNIRYLLCNAD<br>EMEPGTYKDRLLMEQLPHLLVEGMLISAFALKAYRGYIFLRGEYIEAAVNLRRAIAE<br>ATEAGLLGKNIMGTGFDFELFVHTGAGRYICGEETALINSLEGRRANPRSKPPFPA<br>TSGAWGKPTCVNNVETLCNVPAILANGVEWYQNISKSKDAGTKLMGFSGRVKNP<br>GLWELPFGTTAREILEDYAGGMRDGLKFKAWQPGGAGTDFLTEAHLDLPMEFESI<br>GKAGSRLGTALAMAVDHEINMVSLVRNLEEFFARESCGWCTPCRDGLPWSVKILR<br>ALERGEGQPGDIETLEQLCRFLGPGKTFCAHAPGAVEPLQSAIKYFREEFEAGIKQ<br>PFSNTHLINGIQPNLLKERW*<br><br>**NuoG**<br>MLMATIHVDGKEYEVNGADNLLEACLSLGLDIPYFCWHPALGSVGACRQCAVKQY<br>QNAEDTRGRLVMSCMTPASDGTFISIDDEEAKQFRESVVEWLMTNHPHDCPVCE<br>EGGNCHLQDMTVMTGHSFRRYRFTKRTHRNQDLGPFISHEMNRCIACYRCVRYY<br>KDYADGTDLGVYGAHDNVYFGRPEDGTLESEFSGNLVEICPTGVFTDKTHSERYN<br>RKWDMQFAPSICQQCSIGCNISPGERYGELRRIENRYNGTVNHYFLCDRGRFGY<br>GYVNLKDRPRQPVQRRGDDFITLNAEQAMQGAADILRQSKKVIGIGSPRASVESN<br>FALRELVGEENFYTGIAHGEQERLQLALKVLREGGIYTPALREIESYDAVLVLGEDV<br>TQTGARVALAVRQAVKGKAREMAAAQKVADWQIAAILNIGQRAKHPLFVTNVDDT<br>RLDDIAAWTYRAPVEDQARLGFAIAHALDNSAPAVDGIEPELQSKIDVIVQALAGAK<br>KPLIISGTNAGSLEVIQAAANVAKALKGRGADVGITMIARSVNSMGLGIMGGGSLE<br>EALTELETGRADAVVVLENDLHRHASAIRVNAALAKAPLVMVVDHQRTAIMENAHL<br>VLSAASFAESDGTVINNEGRAQRFFQVYDPAYYDSKTVMLESWRWLHSLHSTLLS<br>REVDWTQLDHVIDAVVAKIPELAGIKDAAPDATFRIRGQKLAREPHRYSGRTAMRA<br>NISVHEPRQPQDIDTMFTFSMEGNNQPTAHRSQVPFAWAPGWNSPQAWNKFQD

EVGGKLRFGDPGVRLFETSENGLDYFTSVPARFQPQDGKWRIAPYYHLFGSDEL
SQRAPVFQSRMPQPYIKLNPADAAKLGVNAGTRVSFSYDGNTVTLPVEIAEGLTA
GQVGLPMGMSGIAPVLAGAHLEDLKEAQQ*

NuoH
MSWISPELIEILLTILKAVVILLVVVTCGAFMSFGERRLLGLFQNRYGPNRVGWGGS
LQLVADMIKMFFKEDWIPKFSDRVIFTLAPMIAFTSLLLAFAIVPVSPGWVVADLNIGI
LFFLMMAGLAVYAVLFAGWSSNNKYSLLGAMRASAQTLSYEVFLGLSLMGVVAQA
GSFNMTDIVNSQAHVWNVIPQFFGFITFAIAGVAVCHRHPFDQPEARQELADGYHI
EYSGMKFGLFFVGEYIGIVTISALMVTLFFGGWQGPLLPPFIWFALKTAFFMMMFIL
IRASLPRPRYDQVMSFGWKICLPLTLINLLVTAAVILWQAQ*

NuoI
MTLKELLVGFGTQVRSIWMIGLHAFAKRETRMYPEEPVYLPPRYRGRIVLTRDPD
GEERCVACNLCAVACPVGCISLQKAETKDGRWYPEFFRINFSRCIFCGLCEEACP
TTAIQLTPDFEMGEYKRQDLVYEKEDLLISGPGKYPEYNFYRMAGMAIDGKDKGE
AENEAKPIDVKSLLP*

NuoJ
MEFAFYICGLIAILATLRVITHTNPVHALLYLIISLLAISGVFFSLGAYFAGALEIIVYAGA
IMVLFVFVVMMLNLGGSEIEQERQWLKPQVWIGPAILSAIMLVVIVYAILGVNDQGI
DGTPISAKAVGITLFGPYVLAVELASMLLLAGLVVAFHVGREERAGEVLSNRKDDS
AKRKTEEHA*

NuoK
MIPLQHGLILAAILFVLGLTGLVIRRNLLFMLIGLEIMINASALAFVVAGSYWGQTDG
QVMYILAISLAAAEASIGLALLLQLHRRRQNLNIDSVSEMRG*

NuoL
MNMLALTIILPLIGFVLLAFSRGRWSENVSAIVGVGSVGLAALVTAFIGVDFFANGE
QTYSQPLWTWMSVGDFNIGFNLVLDGLSLTMLSVVTGVGFLIHMYASWYMRGEE
GYSRFFAYTNLFIASMVVLVLADNLLLMYLGWEGVGLCSYLLIGFYYTDPKNGAAA
MKAFVVTRVGDVFLAFALFILYNELGTLNFREMVELAPAHFADGNNMLMWATLMLL
GGAVGKSAQLPLQTWLADAMAGPTPVSALIHAATMVTAGVYLIARTHGLFLMTPE
VLHLVGIVGAVTLLLAGFAALVQTDIKRVLAYSTMSQIGYMFLALGVQAWDAAIFHL
MTHAFFKALLFLASGSVILACHHEQNIFKMGGLRKSIPLVYLCFLVGGAALSALPLV
TAGFFSKDEILAGAMANGHINLMVAGLVGAFMTSLYTFRMIFIVFHGKEQIHAHAVK
GVTHSLPLIVLLILSTFVGALIVPPLQGVLPQTTELAHGSMLTLEITSGVVAVVGILLA
AWLWLGKRTLVTSIANSAPGRLLGTWWYNAWGFDWLYDKVFVKPFLGIAWLLKR
DPLNSMMNIPAVLSRFAGKGLLLSENGYLRWYVASMSIGAVVVLALLMVLR*

NuoM
MLLPWLILIPFIGGFLCWQTERFGVKVPRWIALITMGLTLALSLQLWLQGGYSLTQS
AGIPQWQSEFDMPWIPRFGISIHLAIDGLSLLMVVLTGLLGVLAVLCSWKEIEKYQG
FFHLNLMWILGGVIGVFLAIDMFLFFFFWEMMLVPMYFLIALWGHKASDGKTRITAA
TKFFIYTQASGLVMLIAILALVFVHYNATGVWTFNYEELLNTPMSSGVEYLLMLGFFI
AFAVKMPVVPLHGWLPDAHSQAPTAGSVDLAGILLKTAAYGLLRFSLPLFPNASAE
FAPIAMWLGVIGIFYGAWMAFAQTDIKRLIAYTSVSHMGFVLIAIYTGSQLAYQGAVI
QMIAHGLSAAGLFILCGQLYERIHTRDMRMMGGLWSKMKWLPALSLFFAVATLGM
PGTGNFVGEFMILFGSFQVVPVITVISTFGLVFASVYSLAMLHRAYFGKAKSQIASQ
ELPGMSLRELFMILLLVVLLVLLGFYPQPILDTSHSAIGNIQQWFVNSVTTTRP*

NuoN
MTITPQNLIALLPLLIVGLTVVVVMLSIAWRRNHFLNATLSVIGLNAALVSLWFVGQA
GAMDVTPLMRVDGFAMLYTGLVLLASLATCTFAYPWLEGYNDNKDEFYLLVLIAAL
GGILLANANHLASLFLGIELISLPLFGLVGYAFRQKRSLEASIKYTILSAAASSFLLFG
MALVYAQSGDLSFVALGKNLGDGMLNEPLLLAGFGLMIVGLGFKLSLVPFHLWTP
DVYQGAPAPVSTFLATASKIAIFGVVMRLFLYAPVGDSEAIRVVLAIIAFASIIFGNLM
ALSQTNIKRLLGYSSISHLGYLLVALIALQTGEMSMEAVGVYLAGYLFSSLGAFGVV
SLMSSPYRGPDADSLFSYRGLFWHRPILAAVMTVMMLSLAGIPMTLGFIGKFYVLA

	VGVQAHLWWLVGAVVVGSAIGLYYYLRVAVSLYLHAPEQPGRDAPSNWQYSAGG<br>IVVLISALLVLVLGVWPQPLISIVRLAMPLM*
E241A^{NuoH}	**NuoA**<br>MSMSTSTEVIAHHWAFAIFLIVAIGLCCLMLVGGWFLGGRARARSKNVPFESGIDS<br>VGSARLRLSAKFYLVAMFFVIFDVEALYLFAWSTSIRESGWVGFVEAAIFIFVLLAG<br>LVYLVRIGALDWTPARSRRERMNPETNSIANRQR*<br><br>**NuoB**<br>MDYTLTRIDPNGENDRYPLQKQEIVTDPLEQEVNKNVFMGKLNDMVNWGRKNSI<br>WPYNFGLSCCYVEMVTSFTAVHDVARFGAEVLRASPRQADLMVVAGTCFTKMA<br>PVIQRLYDQMLEPKWVISMGACANSGGMYDIYSVVQGVDKFIPVDVYIPGCPPRP<br>EAYMQALMLLQESIGKERRPLSWVVGDQGVYRANMQSERERKRGERIAVTNLRT<br>PDEI*<br><br>**NuoCD**<br>MVNNMTDLTAQEPAWQTRDHLDDPVIGELRNRFGPDAFTVQATRTGVPVVWIKR<br>EQLLEVGDFLKKLPKPYVMLFDLHGMDERLRTHREGLPAADFSVFYHLISIDRNRD<br>IMLKVALAENDLHVPTFTKLFPNANWYERETWDLFGITFDGHPNLRRIMMPQTWK<br>GHPLRKDYPARATEFSPFELTKAKQDLEMEALTFKPEEWGMKRGTENEDFMFLNL<br>GPNHPSAHGAFRIVLQLDGEEIVDCVPDIGYHHRGAEKMGERQSWHSYIPYTDRI<br>EYLGGCVNEMPYVLAVEKLAGITVPDRVNVIRVMLSELFRINSHLLYISTFIQDVGA<br>MTPVFFAFTDRQKIYDLVEAITGFRMHPAWFRIGGVAHDLPRGWDRLLREFLDWM<br>PKRLASYEKAALQNTILKGRSQGVAAYGAKEALEWGTTGAGLRATGIDFDVRKAR<br>PYSGYENFDFEIPVGGGVSDCYTRVMLKVEELRQSLRILEQCLNNMPEGPFKADH<br>PLTTPPPKERTLQHIETLITHFLQVSWGPVMPANESFQMIEATKGINSYYLTSDGST<br>MSYRTRVRTPSFAHLQQIPAAIRGSLVSDLIVYLGSIDFVMSDVDR*<br><br>**NuoE**<br>MHENQQPQTEAFELSAAEREAIEHEMHHYEDPRAASIEALKIVQKQRGWVPDGAI<br>HAIADVLGIPASDVEGVATFYSQIFRQPVGRHVIRYCDSVVCHINGYQGIQAALEKK<br>LNIKPGQTTFDGRFTLLPTCCLGNCDKGPNMMIDEDTHAHLTPEAIPELLERYK*<br><br>**NuoF**<br>MRGSHHHHHHTDPALRAKNIIRTPETHPLTWRLRDDKQPVWLDEYRSKNGYEGA<br>RKALTGLSPDEIVNQVKDAGLKGRGGAGFSTGLKWSLMPKDESMNIRYLLCNAD<br>EMEPGTYKDRLLMEQLPHLLVEGMLISAFALKAYRGYIFLRGEYIEAAVNLRRAIAE<br>ATEAGLLGKNIMGTGFDFELFVHTGAGRYICGEETALINSLEGRRANPRSKPPFPA<br>TSGAWGKPTCVNNVETLCNVPAILANGVEWYQNISKSKDAGTKLMGFSGRVKNP<br>GLWELPFGTTAREILEDYAGGMRDGLKFKAWQPGGAGTDFLTEAHLDLPMEFESI<br>GKAGSRLGTALAMAVDHEINMVSLVRNLEEFFARESCGWCTPCRDGLPWSVKILR<br>ALERGEGQPGDIETLEQLCRFLGPGKTFCAHAPGAVEPLQSAIKYFREEFEAGIKQ<br>PFSNTHLINGIQPNLLKERW*<br><br>**NuoG**<br>MLMATIHVDGKEYEVNGADNLLEACLSLGLDIPYFCWHPALGSVGACRQCAVKQY<br>QNAEDTRGRLVMSCMTPASDGTFISIDDEEAKQFRESVVEWLMTNHPHDCPVCE<br>EGGNCHLQDMTVMTGHSFRRYRFTKRTHRNQDLGPFISHEMNRCIACYRCVRYY<br>KDYADGTDLGVYGAHDNVYFGRPEDGTLESEFSGNLVEICPTGVFTDKTHSERYN<br>RKWDMQFAPSICQQCSIGCNISPGERYGELRRIENRYNGTVNHYFLCDRGRFGY<br>GYVNLKDRPRQPVQRRGDDFITLNAEQAMQGAADILRQSKKVIGIGSPRASVESN<br>FALRELVGEENFYTGIAHGEQERLQLALKVLREGGIYTPALREIESYDAVLVLGEDV<br>TQTGARVALAVRQAVKGKAREMAAAQKVADWQIAAILNIGQRAKHPLFVTNVDDT<br>RLDDIAAWTYRAPVEDQARLGFAIAHALDNSAPAVDGIEPELQSKIDVIVQALAGAK<br>KPLIISGTNAGSLEVIQAAANVAKALKGRGADVGITMIARSVNSMGLGIMGGGSLE<br>EALTELETGRADAVVVLENDLHRHASAIRVNAALAKAPLVMVVDHQRTAIMENAHL<br>VLSAASFAESDGTVINNEGRAQRFFQVYDPAYYDSKTVMLESWRWLHSLHSTLLS<br>REVDWTQLDHVIDAVVAKIPELAGIKDAAPDATFRIRGQKLAREPHRYSGRTAMRA<br>NISVHEPRQPQDIDTMFTFSMEGNNQPTAHRSQVPFAWAPGWNSPQAWNKFQD

	EVGGKLRFGDPGVRLFETSENGLDYFTSVPARFQPQDGKWRIAPYYHLFGSDEL<br>SQRAPVFQSRMPQPYIKLNPADAAKLGVNAGTRVSFSYDGNTVTLPVEIAEGLTA<br>GQVGLPMGMSGIAPVLAGAHLEDLKEAQQ*<br><br>**NuoH**<br>MSWISPELIEILLTILKAVVILLVVVTCGAFMSFGERRLLGLFQNRYGPNRVGWGGS<br>LQLVADMIKMFFKEDWIPKFSDRVIFTLAPMIAFTSLLLAFAIVPVSPGWVVADLNIGI<br>LFFLMMAGLAVYAVLFAGWSSNNKYSLLGAMRASAQTLSYEVFLGLSLMGVVAQA<br>GSFNMTDIVNSQAHVWNVIPQFFGFITFAIAGVAVCHRHPFDQPEAEQELADGYHI<br>EYSGMKFGLFFVGAYIGIVTISALMVTLFFGGWQGPLLPPFIWFALKTAFFMMMFILI<br>RASLPRPRYDQVMSFGWKICLPLTLINLLVTAAVILWQAQ*<br><br>**NuoI**<br>MTLKELLVGFGTQVRSIWMIGLHAFAKRETRMYPEEPVYLPPRYRGRIVLTRDPD<br>GEERCVACNLCAVACPVGCISLQKAETKDGRWYPEFFRINFSRCIFCGLCEEACP<br>TTAIQLTPDFEMGEYKRQDLVYEKEDLLISGPGKYPEYNFYRMAGMAIDGKDKGE<br>AENEAKPIDVKSLLP*<br><br>**NuoJ**<br>MEFAFYICGLIAILATLRVITHTNPVHALLYLIISLLAISGVFFSLGAYFAGALEIIVYAGA<br>IMVLFVFVVMMLNLGGSEIEQERQWLKPQVWIGPAILSAIMLVVIVYAILGVNDQGI<br>DGTPISAKAVGITLFGPYVLAVELASMLLLAGLVVAFHVGREERAGEVLSNRKDDS<br>AKRKTEEHA*<br><br>**NuoK**<br>MIPLQHGLILAAILFVLGLTGLVIRRNLLFMLIGLEIMINASALAFVVAGSYWGQTDG<br>QVMYILAISLAAAEASIGLALLLQLHRRRQNLNIDSVSEMRG*<br><br>**NuoL**<br>MNMLALTIILPLIGFVLLAFSRGRWSENVSAIVGVGSVGLAALVTAFIGVDFFANGE<br>QTYSQPLWTWMSVGDFNIGFNLVLDGLSLTMLSVVTGVGFLIHMYASWYMRGEE<br>GYSRFFAYTNLFIASMVVLVLADNLLLMYLGWEGVGLCSYLLIGFYYTDPKNGAAA<br>MKAFVVTRVGDVFLAFALFILYNELGTLNFREMVELAPAHFADGNNMLMWATLMLL<br>GGAVGKSAQLPLQTWLADAMAGPTPVSALIHAATMVTAGVYLIARTHGLFLMTPE<br>VLHLVGIVGAVTLLLAGFAALVQTDIKRVLAYSTMSQIGYMFLALGVQAWDAAIFHL<br>MTHAFFKALLFLASGSVILACHHEQNIFKMGGLRKSIPLVYLCFLVGGAALSALPLV<br>TAGFFSKDEILAGAMANGHINLMVAGLVGAFMTSLYTFRMIFIVFHGKEQIHAHAVK<br>GVTHSLPLIVLLILSTFVGALIVPPLQGVLPQTTELAHGSMLTLEITSGVVAVVGILLA<br>AWLWLGKRTLVTSIANSAPGRLLGTWWYNAWGFDWLYDKVFVKPFLGIAWLLKR<br>DPLNSMMNIPAVLSRFAGKGLLLSENGYLRWYVASMSIGAVVVLALLMVLR*<br><br>**NuoM**<br>MLLPWLILIPFIGGFLCWQTERFGVKVPRWIALITMGLTLALSLQLWLQGGYSLTQS<br>AGIPQWQSEFDMPWIPRFGISIHLAIDGLSLLMVVLTGLLGVLAVLCSWKEIEKYQG<br>FFHLNLMWILGGVIGVFLAIDMFLFFFFWEMMLVPMYFLIALWGHKASDGKTRITAA<br>TKFFIYTQASGLVMLIAILALVFVHYNATGVWTFNYEELLNTPMSSGVEYLLMLGFFI<br>AFAVKMPVVPLHGWLPDAHSQAPTAGSVDLAGILLKTAAYGLLRFSLPLFPNASAE<br>FAPIAMWLGVIGIFYGAWMAFAQTDIKRLIAYTSVSHMGFVLIAIYTGSQLAYQGAVI<br>QMIAHGLSAAGLFILCGQLYERIHTRDMRMMGGLWSKMKWLPALSLFFAVATLGM<br>PGTGNFVGEFMILFGSFQVVPVITVISTFGLVFASVYSLAMLHRAYFGKAKSQIASQ<br>ELPGMSLRELFMILLLVVLLVLLGFYPQPILDTSHSAIGNIQQWFVNSVTTTRP*<br><br>**NuoN**<br>MTITPQNLIALLPLLIVGLTVVVVMLSIAWRRNHFLNATLSVIGLNAALVSLWFVGQA<br>GAMDVTPLMRVDGFAMLYTGLVLLASLATCTFAYPWLEGYNDNKDEFYLLVLIAAL<br>GGILLANANHLASLFLGIELISLPLFGLVGYAFRQKRSLEASIKYTILSAAASSFLLFG<br>MALVYAQSGDLSFVALGKNLGDGMLNEPLLLAGFGLMIVGLGFKLSLVPFHLWTP<br>DVYQGAPAPVSTFLATASKIAIFGVVMRLFLYAPVGDSEAIRVVLAIIAFASIIFGNLM<br>ALSQTNIKRLLGYSSISHLGYLLVALIALQTGEMSMEAVGVYLAGYLFSSLGAFGVV<br>SLMSSPYRGPDADSLFSYRGLFWHRPILAAVMTVMMLSLAGIPMTLGFIGKFYVLA

	VGVQAHLWWLVGAVVVGSAIGLYYYLRVAVSLYLHAPEQPGRDAPSNWQYSAGG IVVLISALLVLVLGVWPQPLISIVRLAMPLM*
E241Q NuoH	**NuoA**<br>MSMSTSTEVIAHHWAFAIFLIVAIGLCCLMLVGGWFLGGRARARSKNVPFESGIDS VGSARLRLSAKFYLVAMFFVIFDVEALYLFAWSTSIRESGWVGFVEAAIFIFVLLAG LVYLVRIGALDWTPARSRRERMNPETNSIANRQR*<br><br>**NuoB**<br>MDYTLTRIDPNGENDRYPLQKQEIVTDPLEQEVNKNVFMGKLNDMVNWGRKNSI WPYNFGLSCCYVEMVTSFTAVHDVARFGAEVLRASPRQADLMVVAGTCFTKMA PVIQRLYDQMLEPKWVISMGACANSGGMYDIYSVVQGVDKFIPVDVYIPGCPPRP EAYMQALMLLQESIGKERRPLSWVVGDQGVYRANMQSERERKRGERIAVTNLRT PDEI*<br><br>**NuoCD**<br>MVNNMTDLTAQEPAWQTRDHLDDPVIGELRNRFGPDAFTVQATRTGVPVVWIKR EQLLEVGDFLKKLPKPYVMLFDLHGMDERLRTHREGLPAADFSVFYHLISIDRNRD IMLKVALAENDLHVPTFTKLFPNANWYERETWDLFGITFDGHPNLRRIMMPQTWK GHPLRKDYPARATEFSPFELTKAKQDLEMEALTFKPEEWGMKRGTENEDFMFLNL GPNHPSAHGAFRIVLQLDGEEIVDCVPDIGYHHRGAEKMGERQSWHSYIPYTDRI EYLGGCVNEMPYVLAVEKLAGITVPDRVNVIRVMLSELFRINSHLLYISTFIQDVGA MTPVFFAFTDRQKIYDLVEAITGFRMHPAWFRIGGVAHDLPRGWDRLLREFLDWM PKRLASYEKAALQNTILKGRSQGVAAYGAKEALEWGTTGAGLRATGIDFDVRKAR PYSGYENFDFEIPVGGGVSDCYTRVMLKVEELRQSLRILEQCLNNMPEGPFKADH PLTTPPPKERTLQHIETLITHFLQVSWGPVMPANESFQMIEATKGINSYYLTSDGST MSYRTRVRTPSFAHLQQIPAAIRGSLVSDLIVYLGSIDFVMSDVDR*<br><br>**NuoE**<br>MHENQQPQTEAFELSAAEREAIEHEMHHYEDPRAASIEALKIVQKQRGWVPDGAI HAIADVLGIPASDVEGVATFYSQIFRQPVGRHVIRYCDSVVCHINGYQGIQAALEKK LNIKPGQTTFDGRFTLLPTCCLGNCDKGPNMMIDEDTHAHLTPEAIPELLERYK*<br><br>**NuoF**<br>MRGSHHHHHHTDPALRAKNIIRTPETHPLTWRLRDDKQPVWLDEYRSKNGYEGA RKALTGLSPDEIVNQVKDAGLKGRGGAGFSTGLKWSLMPKDESMNIRYLLCNAD EMEPGTYKDRLLMEQLPHLLVEGMLISAFALKAYRGYIFLRGEYIEAAVNLRRAIAE ATEAGLLGKNIMGTGFDFELFVHTGAGRYICGEETALINSLEGRRANPRSKPPFPA TSGAWGKPTCVNNVETLCNVPAILANGVEWYQNISKSKDAGTKLMGFSGRVKNP GLWELPFGTTAREILEDYAGGMRDGLKFKAWQPGGAGTDFLTEAHLDLPMEFESI GKAGSRLGTALAMAVDHEINMVSLVRNLEEFFARESCGWCTPCRDGLPWSVKILR ALERGEGQPGDIETLEQLCRFLGPGKTFCAHAPGAVEPLQSAIKYFREEFEAGIKQ PFSNTHLINGIQPNLLKERW*<br><br>**NuoG**<br>MLMATIHVDGKEYEVNGADNLLEACLSLGLDIPYFCWHPALGSVGACRQCAVKQY QNAEDTRGRLVMSCMTPASDGTFISIDDEEAKQFRESVVEWLMTNHPHDCPVCE EGGNCHLQDMTVMTGHSFRRYRFTKRTHRNQDLGPFISHEMNRCIACYRCVRYY KDYADGTDLGVYGAHDNVYFGRPEDGTLESEFSGNLVEICPTGVFTDKTHSERYN RKWDMQFAPSICQQCSIGCNISPGERYGELRRIENRYNGTVNHYFLCDRGRFGY GYVNLKDRPRQPVQRRGDDFITLNAEQAMQGAADILRQSKKVIGIGSPRASVESN FALRELVGEENFYTGIAHGEQERLQLALKVLREGGIYTPALREIESYDAVLVLGEDV TQTGARVALAVRQAVKGKAREMAAAQKVADWQIAAILNIGQRAKHPLFVTNVDDT RLDDIAAWTYRAPVEDQARLGFAIAHALDNSAPAVDGIEPELQSKIDVIVQALAGAK KPLIISGTNAGSLEVIQAAANVAKALKGRGADVGITMIARSVNSMGLGIMGGGSLE EALTELETGRADAVVVLENDLHRHASAIRVNAALAKAPLVMVVDHQRTAIMENAHL VLSAASFAESDGTVINNEGRAQRFFQVYDPAYYDSKTVMLESWRWLHSLHSTLLS REVDWTQLDHVIDAVVAKIPELAGIKDAAPDATFRIRGQKLAREPHRYSGRTAMRA NISVHEPRQPQDIDTMFTFSMEGNNQPTAHRSQVPFAWAPGWNSPQAWNKFQD

EVGGKLRFGDPGVRLFETSENGLDYFTSVPARFQPQDGKWRIAPYYHLFGSDEL
SQRAPVFQSRMPQPYIKLNPADAAKLGVNAGTRVSFSYDGNTVTLPVEIAEGLTA
GQVGLPMGMSGIAPVLAGAHLEDLKEAQQ*

NuoH
MSWISPELIEILLTILKAVVILLVVVTCGAFMSFGERRLLGLFQNRYGPNRVGWGGS
LQLVADMIKMFFKEDWIPKFSDRVIFTLAPMIAFTSLLLAFAIVPVSPGWVVADLNIGI
LFFLMMAGLAVYAVLFAGWSSNNKYSLLGAMRASAQTLSYEVFLGLSLMGVVAQA
GSFNMTDIVNSQAHVWNVIPQFFGFITFAIAGVAVCHRHPFDQPEAEQELADGYHI
EYSGMKFGLFFVGQYIGIVTISALMVTLFFGGWQGPLLPPFIWFALKTAFFMMMFIL
IRASLPRPRYDQVMSFGWKICLPLTLINLLVTAAVILWQAQ*

NuoI
MTLKELLVGFGTQVRSIWMIGLHAFAKRETRMYPEEPVYLPPRYRGRIVLTRDPD
GEERCVACNLCAVACPVGCISLQKAETKDGRWYPEFFRINFSRCIFCGLCEEACP
TTAIQLTPDFEMGEYKRQDLVYEKEDLLISGPGKYPEYNFYRMAGMAIDGKDKGE
AENEAKPIDVKSLLP*

NuoJ
MEFAFYICGLIAILATLRVITHTNPVHALLYLIISLLAISGVFFSLGAYFAGALEIIVYAGA
IMVLFVFVVMMLNLGGSEIEQERQWLKPQVWIGPAILSAIMLVVIVYAILGVNDQGI
DGTPISAKAVGITLFGPYVLAVELASMLLLAGLVVAFHVGREERAGEVLSNRKDDS
AKRKTEEHA*

NuoK
MIPLQHGLILAAILFVLGLTGLVIRRNLLFMLIGLEIMINASALAFVVAGSYWGQTDG
QVMYILAISLAAAEASIGLALLLQLHRRRQNLNIDSVSEMRG*

NuoL
MNMLALTIILPLIGFVLLAFSRGRWSENVSAIVGVGSVGLAALVTAFIGVDFFANGE
QTYSQPLWTWMSVGDFNIGFNLVLDGLSLTMLSVVTGVGFLIHMYASWYMRGEE
GYSRFFAYTNLFIASMVVLVLADNLLLMYLGWEGVGLCSYLLIGFYYTDPKNGAAA
MKAFVVTRVGDVFLAFALFILYNELGTLNFREMVELAPAHFADGNNMLMWATLMLL
GGAVGKSAQLPLQTWLADAMAGPTPVSALIHAATMVTAGVYLIARTHGLFLMTPE
VLHLVGIVGAVTLLLAGFAALVQTDIKRVLAYSTMSQIGYMFLALGVQAWDAAIFHL
MTHAFFKALLFLASGSVILACHHEQNIFKMGGLRKSIPLVYLCFLVGGAALSALPLV
TAGFFSKDEILAGAMANGHINLMVAGLVGAFMTSLYTFRMIFIVFHGKEQIHAHAVK
GVTHSLPLIVLLILSTFVGALIVPPLQGVLPQTTELAHGSMLTLEITSGVVAVVGILLA
AWLWLGKRTLVTSIANSAPGRLLGTWWYNAWGFDWLYDKVFVKPFLGIAWLLKR
DPLNSMMNIPAVLSRFAGKGLLLSENGYLRWYVASMSIGAVVVLALLMVLR*

NuoM
MLLPWLILIPFIGGFLCWQTERFGVKVPRWIALITMGLTLALSLQLWLQGGYSLTQS
AGIPQWQSEFDMPWIPRFGISIHLAIDGLSLLMVVLTGLLGVLAVLCSWKEIEKYQG
FFHLNLMWILGGVIGVFLAIDMFLFFFFWEMMLVPMYFLIALWGHKASDGKTRITAA
TKFFIYTQASGLVMLIAILALVFVHYNATGVWTFNYEELLNTPMSSGVEYLLMLGFFI
AFAVKMPVVPLHGWLPDAHSQAPTAGSVDLAGILLKTAAYGLLRFSLPLFPNASAE
FAPIAMWLGVIGIFYGAWMAFAQTDIKRLIAYTSVSHMGFVLIAIYTGSQLAYQGAVI
QMIAHGLSAAGLFILCGQLYERIHTRDMRMMGGLWSKMKWLPALSLFFAVATLGM
PGTGNFVGEFMILFGSFQVVPVITVISTFGLVFASVYSLAMLHRAYFGKAKSQIASQ
ELPGMSLRELFMILLLVVLLVLLGFYPQPILDTSHSAIGNIQQWFVNSVTTTRP*

NuoN
MTITPQNLIALLPLLIVGLTVVVVMLSIAWRRNHFLNATLSVIGLNAALVSLWFVGQA
GAMDVTPLMRVDGFAMLYTGLVLLASLATCTFAYPWLEGYNDNKDEFYLLVLIAAL
GGILLANANHLASLFLGIELISLPLFGLVGYAFRQKRSLEASIKYTILSAAASSFLLFG
MALVYAQSGDLSFVALGKNLGDGMLNEPLLLAGFGLMIVGLGFKLSLVPFHLWTP
DVYQGAPAPVSTFLATASKIAIFGVVMRLFLYAPVGDSEAIRVVLAIIAFASIIFGNLM
ALSQTNIKRLLGYSSISHLGYLLVALIALQTGEMSMEAVGVYLAGYLFSSLGAFGVV
SLMSSPYRGPDADSLFSYRGLFWHRPILAAVMTVMMLSLAGIPMTLGFIGKFYVLA

	VGVQAHLWWLVGAVVVGSAIGLYYYLRVAVSLYLHAPEQPGRDAPSNWQYSAGG<br>IVVLISALLVLVLGVWPQPLISIVRLAMPLM*
$I63A^{NuoJ}$	**NuoA**<br>MSMSTSTEVIAHHWAFAIFLIVAIGLCCLMLVGGWFLGGRARARSKNVPFESGIDS<br>VGSARLRLSAKFYLVAMFFVIFDVEALYLFAWSTSIRESGWVGFVEAAIFIFVLLAG<br>LVYLVRIGALDWTPARSRRERMNPETNSIANRQR*<br><br>**NuoB**<br>MDYTLTRIDPNGENDRYPLQKQEIVTDPLEQEVNKNVFMGKLNDMVNWGRKNSI<br>WPYNFGLSCCYVEMVTSFTAVHDVARFGAEVLRASPRQADLMVVAGTCFTKMA<br>PVIQRLYDQMLEPKWVISMGACANSGGMYDIYSVVQGVDKFIPVDVYIPGCPPRP<br>EAYMQALMLLQESIGKERRPLSWVVGDQGVYRANMQSERERKRGERIAVTNLRT<br>PDEI*<br><br>**NuoCD**<br>MVNNMTDLTAQEPAWQTRDHLDDPVIGELRNRFGPDAFTVQATRTGVPVVWIKR<br>EQLLEVGDFLKKLPKPYVMLFDLHGMDERLRTHREGLPAADFSVFYHLISIDRNRD<br>IMLKVALAENDLHVPTFTKLFPNANWYERETWDLFGITFDGHPNLRRIMMPQTWK<br>GHPLRKDYPARATEFSPFELTKAKQDLEMEALTFKPEEWGMKRGTENEDFMFLNL<br>GPNHPSAHGAFRIVLQLDGEEIVDCVPDIGYHHRGAEKMGERQSWHSYIPYTDRI<br>EYLGGCVNEMPYVLAVEKLAGITVPDRVNVIRVMLSELFRINSHLLYISTFIQDVGA<br>MTPVFFAFTDRQKIYDLVEAITGFRMHPAWFRIGGVAHDLPRGWDRLLREFLDWM<br>PKRLASYEKAALQNTILKGRSQGVAAYGAKEALEWGTTGAGLRATGIDFDVRKAR<br>PYSGYENFDFEIPVGGGVSDCYTRVMLKVEELRQSLRILEQCLNNMPEGPFKADH<br>PLTTPPPKERTLQHIETLITHFLQVSWGPVMPANESFQMIEATKGINSYYLTSDGST<br>MSYRTRVRTPSFAHLQQIPAAIRGSLVSDLIVYLGSIDFVMSDVDR*<br><br>**NuoE**<br>MHENQQPQTEAFELSAAEREAIEHEMHHYEDPRAASIEALKIVQKQRGWVPDGAI<br>HAIADVLGIPASDVEGVATFYSQIFRQPVGRHVIRYCDSVVCHINGYQGIQAALEKK<br>LNIKPGQTTFDGRFTLLPTCCLGNCDKGPNMMIDEDTHAHLTPEAIPELLERYK*<br><br>**NuoF**<br>MRGSHHHHHHTDPALRAKNIIRTPETHPLTWRLRDDKQPVWLDEYRSKNGYEGA<br>RKALTGLSPDEIVNQVKDAGLKGRGGAGFSTGLKWSLMPKDESMNIRYLLCNAD<br>EMEPGTYKDRLLMEQLPHLLVEGMLISAFALKAYRGYIFLRGEYIEAAVNLRRAIAE<br>ATEAGLLGKNIMGTGFDFELFVHTGAGRYICGEETALINSLEGRRANPRSKPPFPA<br>TSGAWGKPTCVNNVETLCNVPAILANGVEWYQNISKSKDAGTKLMGFSGRVKNP<br>GLWELPFGTTAREILEDYAGGMRDGLKFKAWQPGGAGTDFLTEAHLDLPMEFESI<br>GKAGSRLGTALAMAVDHEINMVSLVRNLEEFFARESCGWCTPCRDGLPWSVKILR<br>ALERGEGQPGDIETLEQLCRFLGPGKTFCAHAPGAVEPLQSAIKYFREEFEAGIKQ<br>PFSNTHLINGIQPNLLKERW*<br><br>**NuoG**<br>MLMATIHVDGKEYEVNGADNLLEACLSLGLDIPYFCWHPALGSVGACRQCAVKQY<br>QNAEDTRGRLVMSCMTPASDGTFISIDDEEAKQFRESVVEWLMTNHPHDCPVCE<br>EGGNCHLQDMTVMTGHSFRRYRFTKRTHRNQDLGPFISHEMNRCIACYRCVRYY<br>KDYADGTDLGVYGAHDNVYFGRPEDGTLESEFSGNLVEICPTGVFTDKTHSERYN<br>RKWDMQFAPSICQQCSIGCNISPGERYGELRRIENRYNGTVNHYFLCDRGRFGY<br>GYVNLKDRPRQPVQRRGDDFITLNAEQAMQGAADILRQSKKVIGIGSPRASVESN<br>FALRELVGEENFYTGIAHGEQERLQLALKVLREGGIYTPALREIESYDAVLVLGEDV<br>TQTGARVALAVRQAVKGKAREMAAAQKVADWQIAAILNIGQRAKHPLFVTNVDDT<br>RLDDIAAWTYRAPVEDQARLGFAIAHALDNSAPAVDGIEPELQSKIDVIVQALAGAK<br>KPLIISGTNAGSLEVIQAAANVAKALKGRGADVGITMIARSVNSMGLGIMGGGSLE<br>EALTELETGRADAVVVLENDLHRHASAIRVNAALAKAPLVMVVDHQRTAIMENAHL<br>VLSAASFAESDGTVINNEGRAQRFFQVYDPAYYDSKTVMLESWRWLHSLHSTLLS<br>REVDWTQLDHVIDAVVAKIPELAGIKDAAPDATFRIRGQKLAREPHRYSGRTAMRA<br>NISVHEPRQPQDIDTMFTFSMEGNNQPTAHRSQVPFAWAPGWNSPQAWNKFQD

EVGGKLRFGDPGVRLFETSENGLDYFTSVPARFQPQDGKWRIAPYYHLFGSDEL
SQRAPVFQSRMPQPYIKLNPADAAKLGVNAGTRVSFSYDGNTVTLPVEIAEGLTA
GQVGLPMGMSGIAPVLAGAHLEDLKEAQQ*

NuoH
MSWISPELIEILLTILKAVVILLVVVTCGAFMSFGERRLLGLFQNRYGPNRVGWGGS
LQLVADMIKMFFKEDWIPKFSDRVIFTLAPMIAFTSLLLAFAIVPVSPGWVVADLNIGI
LFFLMMAGLAVYAVLFAGWSSNNKYSLLGAMRASAQTLSYEVFLGLSLMGVVAQA
GSFNMTDIVNSQAHVWNVIPQFFGFITFAIAGVAVCHRHPFDQPEAEQELADGYHI
EYSGMKFGLFFVGEYIGIVTISALMVTLFFGGWQGPLLPPFIWFALKTAFFMMMFIL
IRASLPRPRYDQVMSFGWKICLPLTLINLLVTAAVILWQAQ*

NuoI
MTLKELLVGFGTQVRSIWMIGLHAFAKRETRMYPEEPVYLPPRYRGRIVLTRDPD
GEERCVACNLCAVACPVGCISLQKAETKDGRWYPEFFRINFSRCIFCGLCEEACP
TTAIQLTPDFEMGEYKRQDLVYEKEDLLISGPGKYPEYNFYRMAGMAIDGKDKGE
AENEAKPIDVKSLLP*

NuoJ
MEFAFYICGLIAILATLRVITHTNPVHALLYLIISLLAISGVFFSLGAYFAGALEIIVYAGA
AMVLFVFVVMMLNLGGSEIEQERQWLKPQVWIGPAILSAIMLVVIVYAILGVNDQGI
DGTPISAKAVGITLFGPYVLAVELASMLLLAGLVVAFHVGREERAGEVLSNRKDDS
AKRKTEEHA*

NuoK
MIPLQHGLILAAILFVLGLTGLVIRRNLLFMLIGLEIMINASALAFVVAGSYWGQTDG
QVMYILAISLAAAEASIGLALLLQLHRRRQNLNIDSVSEMRG*

NuoL
MNMLALTIILPLIGFVLLAFSRGRWSENVSAIVGVGSVGLAALVTAFIGVDFFANGE
QTYSQPLWTWMSVGDFNIGFNLVLDGLSLTMLSVVTGVGFLIHMYASWYMRGEE
GYSRFFAYTNLFIASMVVLVLADNLLLMYLGWEGVGLCSYLLIGFYYTDPKNGAAA
MKAFVVTRVGDVFLAFALFILYNELGTLNFREMVELAPAHFADGNNMLMWATLMLL
GGAVGKSAQLPLQTWLADAMAGPTPVSALIHAATMVTAGVYLIARTHGLFLMTPE
VLHLVGIVGAVTLLLAGFAALVQTDIKRVLAYSTMSQIGYMFLALGVQAWDAAIFHL
MTHAFFKALLFLASGSVILACHHEQNIFKMGGLRKSIPLVYLCFLVGGAALSALPLV
TAGFFSKDEILAGAMANGHINLMVAGLVGAFMTSLYTFRMIFIVFHGKEQIHAHAVK
GVTHSLPLIVLLILSTFVGALIVPPLQGVLPQTTELAHGSMLTLEITSGVVAVVGILLA
AWLWLGKRTLVTSIANSAPGRLLGTWWYNAWGFDWLYDKVFVKPFLGIAWLLKR
DPLNSMMNIPAVLSRFAGKGLLLSENGYLRWYVASMSIGAVVVLALLMVLR*

NuoM
MLLPWLILIPFIGGFLCWQTERFGVKVPRWIALITMGLTLALSLQLWLQGGYSLTQS
AGIPQWQSEFDMPWIPRFGISIHLAIDGLSLLMVVLTGLLGVLAVLCSWKEIEKYQG
FFHLNLMWILGGVIGVFLAIDMFLFFFFWEMMLVPMYFLIALWGHKASDGKTRITAA
TKFFIYTQASGLVMLIAILALVFVHYNATGVWTFNYEELLNTPMSSGVEYLLMLGFFI
AFAVKMPVVPLHGWLPDAHSQAPTAGSVDLAGILLKTAAYGLLRFSLPLFPNASAE
FAPIAMWLGVIGIFYGAWMAFAQTDIKRLIAYTSVSHMGFVLIAIYTGSQLAYQGAVI
QMIAHGLSAAGLFILCGQLYERIHTRDMRMMGGLWSKMKWLPALSLFFAVATLGM
PGTGNFVGEFMILFGSFQVVPVITVISTFGLVFASVYSLAMLHRAYFGKAKSQIASQ
ELPGMSLRELFMILLLVVLLVLLGFYPQPILDTSHSAIGNIQQWFVNSVTTTRP*

NuoN
MTITPQNLIALLPLLIVGLTVVVVMLSIAWRRNHFLNATLSVIGLNAALVSLWFVGQA
GAMDVTPLMRVDGFAMLYTGLVLLASLATCTFAYPWLEGYNDNKDEFYLLVLIAAL
GGILLANANHLASLFLGIELISLPLFGLVGYAFRQKRSLEASIKYTILSAAASSFLLFG
MALVYAQSGDLSFVALGKNLGDGMLNEPLLLAGFGLMIVGLGFKLSLVPFHLWTP
DVYQGAPAPVSTFLATASKIAIFGVVMRLFLYAPVGDSEAIRVVLAIIAFASIIFGNLM
ALSQTNIKRLLGYSSISHLGYLLVALIALQTGEMSMEAVGVYLAGYLFSSLGAFGVV
SLMSSPYRGPDADSLFSYRGLFWHRPILAAVMTVMMLSLAGIPMTLGFIGKFYVLA

	VGVQAHLWWLVGAVVVGSAIGLYYYLRVAVSLYLHAPEQPGRDAPSNWQYSAGG<br>IVVLISALLVLVLGVWPQPLISIVRLAMPLM*
I63M[NuoJ]	**NuoA**<br>MSMSTSTEVIAHHWAFAIFLIVAIGLCCLMLVGGWFLGGRARARSKNVPFESGIDS<br>VGSARLRLSAKFYLVAMFFVIFDVEALYLFAWSTSIRESGWVGFVEAAIFIFVLLAG<br>LVYLVRIGALDWTPARSRRERMNPETNSIANRQR*<br><br>**NuoB**<br>MDYTLTRIDPNGENDRYPLQKQEIVTDPLEQEVNKNVFMGKLNDMVNWGRKNSI<br>WPYNFGLSCCYVEMVTSFTAVHDVARFGAEVLRASPRQADLMVVAGTCFTKMA<br>PVIQRLYDQMLEPKWVISMGACANSGGMYDIYSVVQGVDKFIPVDVYIPGCPPRP<br>EAYMQALMLLQESIGKERRPLSWVVGDQGVYRANMQSERERKRGERIAVTNLRT<br>PDEI*<br><br>**NuoCD**<br>MVNNMTDLTAQEPAWQTRDHLDDPVIGELRNRFGPDAFTVQATRTGVPVVWIKR<br>EQLLEVGDFLKKLPKPYVMLFDLHGMDERLRTHREGLPAADFSVFYHLISIDRNRD<br>IMLKVALAENDLHVPTFTKLFPNANWYERETWDLFGITFDGHPNLRRIMMPQTWK<br>GHPLRKDYPARATEFSPFELTKAKQDLEMEALTFKPEEWGMKRGTENEDFMFLNL<br>GPNHPSAHGAFRIVLQLDGEEIVDCVPDIGYHHRGAEKMGERQSWHSYIPYTDRI<br>EYLGGCVNEMPYVLAVEKLAGITVPDRVNVIRVMLSELFRINSHLLYISTFIQDVGA<br>MTPVFFAFTDRQKIYDLVEAITGFRMHPAWFRIGGVAHDLPRGWDRLLREFLDWM<br>PKRLASYEKAALQNTILKGRSQGVAAYGAKEALEWGTTGAGLRATGIDFDVRKAR<br>PYSGYENFDFEIPVGGGVSDCYTRVMLKVEELRQSLRILEQCLNNMPEGPFKADH<br>PLTTPPPKERTLQHIETLITHFLQVSWGPVMPANESFQMIEATKGINSYYLTSDGST<br>MSYRTRVRTPSFAHLQQIPAAIRGSLVSDLIVYLGSIDFVMSDVDR*<br><br>**NuoE**<br>MHENQQPQTEAFELSAAEREAIEHEMHHYEDPRAASIEALKIVQKQRGWVPDGAI<br>HAIADVLGIPASDVEGVATFYSQIFRQPVGRHVIRYCDSVVCHINGYQGIQAALEKK<br>LNIKPGQTTFDGRFTLLPTCCLGNCDKGPNMMIDEDTHAHLTPEAIPELLERYK*<br><br>**NuoF**<br>MRGSHHHHHHTDPALRAKNIIRTPETHPLTWRLRDDKQPVWLDEYRSKNGYEGA<br>RKALTGLSPDEIVNQVKDAGLKGRGGAGFSTGLKWSLMPKDESMNIRYLLCNAD<br>EMEPGTYKDRLLMEQLPHLLVEGMLISAFALKAYRGYIFLRGEYIEAAVNLRRAIAE<br>ATEAGLLGKNIMGTGFDFELFVHTGAGRYICGEETALINSLEGRRANPRSKPPFPA<br>TSGAWGKPTCVNNVETLCNVPAILANGVEWYQNISKSKDAGTKLMGFSGRVKNP<br>GLWELPFGTTAREILEDYAGGMRDGLKFKAWQPGGAGTDFLTEAHLDLPMEFESI<br>GKAGSRLGTALAMAVDHEINMVSLVRNLEEFFARESCGWCTPCRDGLPWSVKILR<br>ALERGEGQPGDIETLEQLCRFLGPGKTFCAHAPGAVEPLQSAIKYFREEFEAGIKQ<br>PFSNTHLINGIQPNLLKERW*<br><br>**NuoG**<br>MLMATIHVDGKEYEVNGADNLLEACLSLGLDIPYFCWHPALGSVGACRQCAVKQY<br>QNAEDTRGRLVMSCMTPASDGTFISIDDEEAKQFRESVVEWLMTNHPHDCPVCE<br>EGGNCHLQDMTVMTGHSFRRYRFTKRTHRNQDLGPFISHEMNRCIACYRCVRYY<br>KDYADGTDLGVYGAHDNVYFGRPEDGTLESEFSGNLVEICPTGVFTDKTHSERYN<br>RKWDMQFAPSICQQCSIGCNISPGERYGELRRIENRYNGTVNHYFLCDRGRFGY<br>GYVNLKDRPRQPVQRRGDDFITLNAEQAMQGAADILRQSKKVIGIGSPRASVESN<br>FALRELVGEENFYTGIAHGEQERLQLALKVLREGGIYTPALREIESYDAVLVLGEDV<br>TQTGARVALAVRQAVKGKAREMAAAQKVADWQIAAILNIGQRAKHPLFVTNVDDT<br>RLDDIAAWTYRAPVEDQARLGFAIAHALDNSAPAVDGIEPELQSKIDVIVQALAGAK<br>KPLIISGTNAGSLEVIQAAANVAKALKGRGADVGITMIARSVNSMGLGIMGGGSLE<br>EALTELETGRADAVVVLENDLHRHASAIRVNAALAKAPLVMVVDHQRTAIMENAHL<br>VLSAASFAESDGTVINNEGRAQRFFQVYDPAYYDSKTVMLESWRLHSLHSTLLS<br>REVDWTQLDHVIDAVVAKIPELAGIKDAAPDATFRIRGQKLAREPHRYSGRTAMRA<br>NISVHEPRQPQDIDTMFTFSMEGNNQPTAHRSQVPFAWAPGWNSPQAWNKFQD

EVGGKLRFGDPGVRLFETSENGLDYFTSVPARFQPQDGKWRIAPYYHLFGSDEL
SQRAPVFQSRMPQPYIKLNPADAAKLGVNAGTRVSFSYDGNTVTLPVEIAEGLTA
GQVGLPMGMSGIAPVLAGAHLEDLKEAQQ*

NuoH
MSWISPELIEILLTILKAVVILLVVVTCGAFMSFGERRLLGLFQNRYGPNRVGWGGS
LQLVADMIKMFFKEDWIPKFSDRVIFTLAPMIAFTSLLLAFAIVPVSPGWVVADLNIGI
LFFLMMAGLAVYAVLFAGWSSNNKYSLLGAMRASAQTLSYEVFLGLSLMGVVAQA
GSFNMTDIVNSQAHVWNVIPQFFGFITFAIAGVAVCHRHPFDQPEAEQELADGYHI
EYSGMKFGLFFVGEYIGIVTISALMVTLFFGGWQGPLLPPFIWFALKTAFFMMMFIL
IRASLPRPRYDQVMSFGWKICLPLTLINLLVTAAVILWQAQ*

NuoI
MTLKELLVGFGTQVRSIWMIGLHAFAKRETRMYPEEPVYLPPRYRGRIVLTRDPD
GEERCVACNLCAVACPVGCISLQKAETKDGRWYPEFFRINFSRCIFCGLCEEACP
TTAIQLTPDFEMGEYKRQDLVYEKEDLLISGPGKYPEYNFYRMAGMAIDGKDKGE
AENEAKPIDVKSLLP*

NuoJ
MEFAFYICGLIAILATLRVITHTNPVHALLYLIISLLAISGVFFSLGAYFAGALEIIVYAGA
MMVLFVFVVMMLNLGGSEIEQERQWLKPQVWIGPAILSAIMLVVIVYAILGVNDQGI
DGTPISAKAVGITLFGPYVLAVELASMLLLAGLVVAFHVGREERAGEVLSNRKDDS
AKRKTEEHA*

NuoK
MIPLQHGLILAAILFVLGLTGLVIRRNLLFMLIGLEIMINASALAFVVAGSYWGQTDG
QVMYILAISLAAAEASIGLALLLQLHRRRQNLNIDSVSEMRG*

NuoL
MNMLALTIILPLIGFVLLAFSRGRWSENVSAIVGVGSVGLAALVTAFIGVDFFANGE
QTYSQPLWTWMSVGDFNIGFNLVLDGLSLTMLSVVTGVGFLIHMYASWYMRGEE
GYSRFFAYTNLFIASMVVLVLADNLLLMYLGWEGVGLCSYLLIGFYYTDPKNGAAA
MKAFVVTRVGDVFLAFALFILYNELGTLNFREMVELAPAHFADGNNMLMWATLMLL
GGAVGKSAQLPLQTWLADAMAGPTPVSALIHAATMVTAGVYLIARTHGLFLMTPE
VLHLVGIVGAVTLLLAGFAALVQTDIKRVLAYSTMSQIGYMFLALGVQAWDAAIFHL
MTHAFFKALLFLASGSVILACHHEQNIFKMGGLRKSIPLVYLCFLVGGAALSALPLV
TAGFFSKDEILAGAMANGHINLMVAGLVGAFMTSLYTFRMIFIVFHGKEQIHAHAVK
GVTHSLPLIVLLILSTFVGALIVPPLQGVLPQTTELAHGSMLTLEITSGVVAVVGILLA
AWLWLGKRTLVTSIANSAPGRLLGTWWYNAWGFDWLYDKVFVKPFLGIAWLLKR
DPLNSMMNIPAVLSRFAGKGLLLSENGYLRWYVASMSIGAVVVLALLMVLR*

NuoM
MLLPWLILIPFIGGFLCWQTERFGVKVPRWIALITMGLTLALSLQLWLQGGYSLTQS
AGIPQWQSEFDMPWIPRFGISIHLAIDGLSLLMVVLTGLLGVLAVLCSWKEIEKYQG
FFHLNLMWILGGVIGVFLAIDMFLFFFFWEMMLVPMYFLIALWGHKASDGKTRITAA
TKFFIYTQASGLVMLIAILALVFVHYNATGVWTFNYEELLNTPMSSGVEYLLMLGFFI
AFAVKMPVVPLHGWLPDAHSQAPTAGSVDLAGILLKTAAYGLLRFSLPLFPNASAE
FAPIAMWLGVIGIFYGAWMAFAQTDIKRLIAYTSVSHMGFVLIAIYTGSQLAYQGAVI
QMIAHGLSAAGLFILCGQLYERIHTRDMRMMGGLWSKMKWLPALSLFFAVATLGM
PGTGNFVGEFMILFGSFQVVPVITVISTFGLVFASVYSLAMLHRAYFGKAKSQIASQ
ELPGMSLRELFMILLLVVLLVLLGFYPQPILDTSHSAIGNIQQWFVNSVTTTRP*

NuoN
MTITPQNLIALLPLLIVGLTVVVVMLSIAWRRNHFLNATLSVIGLNAALVSLWFVGQA
GAMDVTPLMRVDGFAMLYTGLVLLASLATCTFAYPWLEGYNDNKDEFYLLVLIAAL
GGILLANANHLASLFLGIELISLPLFGLVGYAFRQKRSLEASIKYTILSAAASSFLLFG
MALVYAQSGDLSFVALGKNLGDGMLNEPLLLAGFGLMIVGLGFKLSLVPFHLWTP
DVYQGAPAPVSTFLATASKIAFGVVMRLFLYAPVGDSEAIRVVLAIIAFASIIFGNLM
ALSQTNIKRLLGYSSISHLGYLLVALIALQTGEMSMEAVGVYLAGYLFSSLGAFGVV
SLMSSPYRGPDADSLFSYRGLFWHRPILAAVMTVMMLSLAGIPMTLGFIGKFYVLA

	VGVQAHLWWLVGAVVVGSAIGLYYYLRVAVSLYLHAPEQPGRDAPSNWQYSAGG<br>IVVLISALLVLVLGVWPQPLISIVRLAMPLM*
D79A^{NuoA}	**NuoA**<br>MSMSTSTEVIAHHWAFAIFLIVAIGLCCLMLVGGWFLGGRARARSKNVPFESGIDS<br>VGSARLRLSAKFYLVAMFFVIFAVEALYLFAWSTSIRESGWVGFVEAAIFIFVLLAG<br>LVYLVRIGALDWTPARSRRERMNPETNSIANRQR*<br><br>**NuoB**<br>MDYTLTRIDPNGENDRYPLQKQEIVTDPLEQEVNKNVFMGKLNDMVNWGRKNSI<br>WPYNFGLSCCYVEMVTSFTAVHDVARFGAEVLRASPRQADLMVVAGTCFTKMA<br>PVIQRLYDQMLEPKWVISMGACANSGGMYDIYSVVQGVDKFIPVDVYIPGCPPRP<br>EAYMQALMLLQESIGKERRPLSWVVGDQGVYRANMQSERERKRGERIAVTNLRT<br>PDEI*<br><br>**NuoCD**<br>MVNNMTDLTAQEPAWQTRDHLDDPVIGELRNRFGPDAFTVQATRTGVPVVWIKR<br>EQLLEVGDFLKKLPKPYVMLFDLHGMDERLRTHREGLPAADFSVFYHLISIDRNRD<br>IMLKVALAENDLHVPTFTKLFPNANWYERETWDLFGITFDGHPNLRRIMMPQTWK<br>GHPLRKDYPARATEFSPFELTKAKQDLEMEALTFKPEEWGMKRGTENEDFMFLNL<br>GPNHPSAHGAFRIVLQLDGEEIVDCVPDIGYHHRGAEKMGERQSWHSYIPYTDRI<br>EYLGGCVNEMPYVLAVEKLAGITVPDRVNVIRVMLSELFRINSHLLYISTFIQDVGA<br>MTPVFFAFTDRQKIYDLVEAITGFRMHPAWFRIGGVAHDLPRGWDRLLREFLDWM<br>PKRLASYEKAALQNTILKGRSQGVAAYGAKEALEWGTTGAGLRATGIDFDVRKAR<br>PYSGYENFDFEIPVGGGVSDCYTRVMLKVEELRQSLRILEQCLNNMPEGPFKADH<br>PLTTPPPKERTLQHIETLITHFLQVSWGPVMPANESFQMIEATKGINSYYLTSDGST<br>MSYRTRVRTPSFAHLQQIPAAIRGSLVSDLIVYLGSIDFVMSDVDR*<br><br>**NuoE**<br>MHENQQPQTEAFELSAAEREAIEHEMHHYEDPRAASIEALKIVQKQRGWVPDGAI<br>HAIADVLGIPASDVEGVATFYSQIFRQPVGRHVIRYCDSVVCHINGYQGIQAALEKK<br>LNIKPGQTTFDGRFTLLPTCCLGNCDKGPNMMIDEDTHAHLTPEAIPELLERYK*<br><br>**NuoF**<br>MRGSHHHHHHTDPALRAKNIIRTPETHPLTWRLRDDKQPVWLDEYRSKNGYEGA<br>RKALTGLSPDEIVNQVKDAGLKGRGGAGFSTGLKWSLMPKDESMNIRYLLCNAD<br>EMEPGTYKDRLLMEQLPHLLVEGMLISAFALKAYRGYIFLRGEYIEAAVNLRRAIAE<br>ATEAGLLGKNIMGTGFDFELFVHTGAGRYICGEETALINSLEGRRANPRSKPPFPA<br>TSGAWGKPTCVNNVETLCNVPAILANGVEWYQNISKSKDAGTKLMGFSGRVKNP<br>GLWELPFGTTAREILEDYAGGMRDGLKFKAWQPGGAGTDFLTEAHLDLPMEFESI<br>GKAGSRLGTALAMAVDHEINMVSLVRNLEEFFARESCGWCTPCRDGLPWSVKILR<br>ALERGEGQPGDIETLEQLCRFLGPGKTFCAHAPGAVEPLQSAIKYFREEFEAGIKQ<br>PFSNTHLINGIQPNLLKERW*<br><br>**NuoG**<br>MLMATIHVDGKEYEVNGADNLLEACLSLGLDIPYFCWHPALGSVGACRQCAVKQY<br>QNAEDTRGRLVMSCMTPASDGTFISIDDEEAKQFRESVVEWLMTNHPHDCPVCE<br>EGGNCHLQDMTVMTGHSFRRYRFTKRTHRNQDLGPFISHEMNRCIACYRCVRYY<br>KDYADGTDLGVYGAHDNVYFGRPEDGTLESEFSGNLVEICPTGVFTDKTHSERYN<br>RKWDMQFAPSICQQCSIGCNISPGERYGELRRIENRYNGTVNHYFLCDRGRFGY<br>GYVNLKDRPRQPVQRRGDDFITLNAEQAMQGAADILRQSKKVIGIGSPRASVESN<br>FALRELVGEENFYTGIAHGEQERLQLALKVLREGGIYTPALREIESYDAVLVLGEDV<br>TQTGARVALAVRQAVKGKAREMAAAQKVADWQIAAILNIGQRAKHPLFVTNVDDT<br>RLDDIAAWTYRAPVEDQARLGFAIAHALDNSAPAVDGIEPELQSKIDVIVQALAGAK<br>KPLIISGTNAGSLEVIQAAANVAKALKGRGADVGITMIARSVNSMGLGIMGGGSLE<br>EALTELETGRADAVVVLENDLHRHASAIRVNAALAKAPLVMVVDHQRTAIMENAHL<br>VLSAASFAESDGTVINNEGRAQRFFQVYDPAYYDSKTVMLESWRWLHSLHSTLLS<br>REVDWTQLDHVIDAVVAKIPELAGIKDAAPDATFRIRGQKLAREPHRYSGRTAMRA<br>NISVHEPRQPQDIDTMFTFSMEGNNQPTAHRSQVPFAWAPGWNSPQAWNKFQD

EVGGKLRFGDPGVRLFETSENGLDYFTSVPARFQPQDGKWRIAPYYHLFGSDEL
SQRAPVFQSRMPQPYIKLNPADAAKLGVNAGTRVSFSYDGNTVTLPVEIAEGLTA
GQVGLPMGMSGIAPVLAGAHLEDLKEAQQ*

NuoH
MSWISPELIEILLTILKAVVILLVVVTCGAFMSFGERRLLGLFQNRYGPNRVGWGGS
LQLVADMIKMFFKEDWIPKFSDRVIFTLAPMIAFTSLLLAFAIVPVSPGWVVADLNIGI
LFFLMMAGLAVYAVLFAGWSSNNKYSLLGAMRASAQTLSYEVFLGLSLMGVVAQA
GSFNMTDIVNSQAHVWNVIPQFFGFITFAIAGVAVCHRHPFDQPEAEQELADGYHI
EYSGMKFGLFFVGEYIGIVTISALMVTLFFGGWQGPLLPPFIWFALKTAFFMMMFIL
IRASLPRPRYDQVMSFGWKICLPLTLINLLVTAAVILWQAQ*

NuoI
MTLKELLVGFGTQVRSIWMIGLHAFAKRETRMYPEEPVYLPPRYRGRIVLTRDPD
GEERCVACNLCAVACPVGCISLQKAETKDGRWYPEFFRINFSRCIFCGLCEEACP
TTAIQLTPDFEMGEYKRQDLVYEKEDLLISGPGKYPEYNFYRMAGMAIDGKDKGE
AENEAKPIDVKSLLP*

NuoJ
MEFAFYICGLIAILATLRVITHTNPVHALLYLIISLLAISGVFFSLGAYFAGALEIIVYAGA
IMVLFVFVVMMLNLGGSEIEQERQWLKPQVWIGPAILSAIMLVVIVYAILGVNDQGI
DGTPISAKAVGITLFGPYVLAVELASMLLLAGLVVAFHVGREERAGEVLSNRKDDS
AKRKTEEHA*

NuoK
MIPLQHGLILAAILFVLGLTGLVIRRNLLFMLIGLEIMINASALAFVVAGSYWGQTDG
QVMYILAISLAAAEASIGLALLLQLHRRRQNLNIDSVSEMRG*

NuoL
MNMLALTIILPLIGFVLLAFSRGRWSENVSAIVGVGSVGLAALVTAFIGVDFFANGE
QTYSQPLWTWMSVGDFNIGFNLVLDGLSLTMLSVVTGVGFLIHMYASWYMRGEE
GYSRFFAYTNLFIASMVVLVLADNLLLMYLGWEGVGLCSYLLIGFYYTDPKNGAAA
MKAFVVTRVGDVFLAFALFILYNELGTLNFREMVELAPAHFADGNNMLMWATLMLL
GGAVGKSAQLPLQTWLADAMAGPTPVSALIHAATMVTAGVYLIARTHGLFLMTPE
VLHLVGIVGAVTLLLAGFAALVQTDIKRVLAYSTMSQIGYMFLALGVQAWDAAIFHL
MTHAFFKALLFLASGSVILACHHEQNIFKMGGLRKSIPLVYLCFLVGGAALSALPLV
TAGFFSKDEILAGAMANGHINLMVAGLVGAFMTSLYTFRMIFIVFHGKEQIHAHAVK
GVTHSLPLIVLLILSTFVGALIVPPLQGVLPQTTELAHGSMLTLEITSGVVAVVGILLA
AWLWLGKRTLVTSIANSAPGRLLGTWWYNAWGFDWLYDKVFVKPFLGIAWLLKR
DPLNSMMNIPAVLSRFAGKGLLLSENGYLRWYVASMSIGAVVVLALLMVLR*

NuoM
MLLPWLILIPFIGGFLCWQTERFGVKVPRWIALITMGLTLALSLQLWLQGGYSLTQS
AGIPQWQSEFDMPWIPRFGISIHLAIDGLSLLMVVLTGLLGVLAVLCSWKEIEKYQG
FFHLNLMWILGGVIGVFLAIDMFLFFFFWEMMLVPMYFLIALWGHKASDGKTRITAA
TKFFIYTQASGLVMLIAILALVFVHYNATGVWTFNYEELLNTPMSSGVEYLLMLGFFI
AFAVKMPVVPLHGWLPDAHSQAPTAGSVDLAGILLKTAAYGLLRFSLPLFPNASAE
FAPIAMWLGVIGIFYGAWMAFAQTDIKRLIAYTSVSHMGFVLIAIYTGSQLAYQGAVI
QMIAHGLSAAGLFILCGQLYERIHTRDMRMMGGLWSKMKWLPALSLFFAVATLGM
PGTGNFVGEFMILFGSFQVVPVITVISTFGLVFASVYSLAMLHRAYFGKAKSQIASQ
ELPGMSLRELFMILLLVVLLVLLGFYPQPILDTSHSAIGNIQQWFVNSVTTTRP*

NuoN
MTITPQNLIALLPLLIVGLTVVVVMLSIAWRRNHFLNATLSVIGLNAALVSLWFVGQA
GAMDVTPLMRVDGFAMLYTGLVLLASLATCTFAYPWLEGYNDNKDEFYLLVLIAAL
GGILLANANHLASLFLGIELISLPLFGLVGYAFRQKRSLEASIKYTILSAAASSFLLFG
MALVYAQSGDLSFVALGKNLGDGMLNEPLLLAGFGLMIVGLGFKLSLVPFHLWTP
DVYQGAPAPVSTFLATASKIAIFGVVMRLFLYAPVGDSEAIRVVLAIIAFASIIFGNLM
ALSQTNIKRLLGYSSISHLGYLLVALIALQTGEMSMEAVGVYLAGYLFSSLGAFGVV
SLMSSPYRGPDADSLFSYRGLFWHRPILAAVMTVMMLSLAGIPMTLGFIGKFYVLA

	VGVQAHLWWLVGAVVVGSAIGLYYYLRVAVSLYLHAPEQPGRDAPSNWQYSAGG<br>IVVLISALLVLVLGVWPQPLISIVRLAMPLM*
E51A^{NuoA}	**NuoA**<br>MSMSTSTEVIAHHWAFAIFLIVAIGLCCLMLVGGWFLGGRARARSKNVPFASGIDS<br>VGSARLRLSAKFYLVAMFFVIFDVEALYLFAWSTSIRESGWVGFVEAAIFIFVLLAG<br>LVYLVRIGALDWTPARSRRERMNPETNSIANRQR*<br><br>**NuoB**<br>MDYTLTRIDPNGENDRYPLQKQEIVTDPLEQEVNKNVFMGKLNDMVNWGRKNSI<br>WPYNFGLSCCYVEMVTSFTAVHDVARFGAEVLRASPRQADLMVVAGTCFTKMA<br>PVIQRLYDQMLEPKWVISMGACANSGGMYDIYSVVQGVDKFIPVDVYIPGCPPRP<br>EAYMQALMLLQESIGKERRPLSWVVGDQGVYRANMQSERERKRGERIAVTNLRT<br>PDEI*<br><br>**NuoCD**<br>MVNNMTDLTAQEPAWQTRDHLDDPVIGELRNRFGPDAFTVQATRTGVPVVWIKR<br>EQLLEVGDFLKKLPKPYVMLFDLHGMDERLRTHREGLPAADFSVFYHLISIDRNRD<br>IMLKVALAENDLHVPTFTKLFPNANWYERETWDLFGITFDGHPNLRRIMMPQTWK<br>GHPLRKDYPARATEFSPFELTKAKQDLEMEALTFKPEEWGMKRGTENEDFMFLNL<br>GPNHPSAHGAFRIVLQLDGEEIVDCVPDIGYHHRGAEKMGERQSWHSYIPYTDRI<br>EYLGGCVNEMPYVLAVEKLAGITVPDRVNVIRVMLSELFRINSHLLYISTFIQDVGA<br>MTPVFFAFTDRQKIYDLVEAITGFRMHPAWFRIGGVAHDLPRGWDRLLREFLDWM<br>PKRLASYEKAALQNTILKGRSQGVAAYGAKEALEWGTTGAGLRATGIDFDVRKAR<br>PYSGYENFDFEIPVGGGVSDCYTRVMLKVEELRQSLRILEQCLNNMPEGPFKADH<br>PLTTPPPKERTLQHIETLITHFLQVSWGPVMPANESFQMIEATKGINSYYLTSDGST<br>MSYRTRVRTPSFAHLQQIPAAIRGSLVSDLIVYLGSIDFVMSDVDR*<br><br>**NuoE**<br>MHENQQPQTEAFELSAAEREAIEHEMHHYEDPRAASIEALKIVQKQRGWVPDGAI<br>HAIADVLGIPASDVEGVATFYSQIFRQPVGRHVIRYCDSVVCHINGYQGIQAALEKK<br>LNIKPGQTTFDGRFTLLPTCCLGNCDKGPNMMIDEDTHAHLTPEAIPELLERYK*<br><br>**NuoF**<br>MRGSHHHHHHTDPALRAKNIIRTPETHPLTWRLRDDKQPVWLDEYRSKNGYEGA<br>RKALTGLSPDEIVNQVKDAGLKGRGGAGFSTGLKWSLMPKDESMNIRYLLCNAD<br>EMEPGTYKDRLLMEQLPHLLVEGMLISAFALKAYRGYIFLRGEYIEAAVNLRRAIAE<br>ATEAGLLGKNIMGTGFDFELFVHTGAGRYICGEETALINSLEGRRANPRSKPPFPA<br>TSGAWGKPTCVNNVETLCNVPAILANGVEWYQNISKSKDAGTKLMGFSGRVKNP<br>GLWELPFGTTAREILEDYAGGMRDGLKFKAWQPGGAGTDFLTEAHLDLPMEFESI<br>GKAGSRLGTALAMAVDHEINMVSLVRNLEEFFARESCGWCTPCRDGLPWSVKILR<br>ALERGEGQPGDIETLEQLCRFLGPGKTFCAHAPGAVEPLQSAIKYFREEFEAGIKQ<br>PFSNTHLINGIQPNLLKERW*<br><br>**NuoG**<br>MLMATIHVDGKEYEVNGADNLLEACLSLGLDIPYFCWHPALGSVGACRQCAVKQY<br>QNAEDTRGRLVMSCMTPASDGTFISIDDEEAKQFRESVVEWLMTNHPHDCPVCE<br>EGGNCHLQDMTVMTGHSFRRYRFTKRTHRNQDLGPFISHEMNRCIACYRCVRYY<br>KDYADGTDLGVYGAHDNVYFGRPEDGTLESEFSGNLVEICPTGVFTDKTHSERYN<br>RKWDMQFAPSICQQCSIGCNISPGERYGELRRIENRYNGTVNHYFLCDRGRFGY<br>GYVNLKDRPRQPVQRRGDDFITLNAEQAMQGAADILRQSKKVIGIGSPRASVESN<br>FALRELVGEENFYTGIAHGEQERLQLALKVLREGGIYTPALREIESYDAVLVLGEDV<br>TQTGARVALAVRQAVKGKAREMAAAQKVADWQIAAILNIGQRAKHPLFVTNVDDT<br>RLDDIAAWTYRAPVEDQARLGFAIAHALDNSAPAVDGIEPELQSKIDVIVQALAGAK<br>KPLIISGTNAGSLEVIQAAANVAKALKGRGADVGITMIARSVNSMGLGIMGGGSLE<br>EALTELETGRADAVVVLENDLHRHASAIRVNAALAKAPLVMVVDHQRTAIMENAHL<br>VLSAASFAESDGTVINNEGRAQRFFQVYDPAYYDSKTVMLESWRWLHSLHSTLLS<br>REVDWTQLDHVIDAVVAKIPELAGIKDAAPDATFRIRGQKLAREPHRYSGRTAMRA<br>NISVHEPRQPQDIDTMFTFSMEGNNQPTAHRSQVPFAWAPGWNSPQAWNKFQD

EVGGKLRFGDPGVRLFETSENGLDYFTSVPARFQPQDGKWRIAPYYHLFGSDEL
SQRAPVFQSRMPQPYIKLNPADAAKLGVNAGTRVSFSYDGNTVTLPVEIAEGLTA
GQVGLPMGMSGIAPVLAGAHLEDLKEAQQ*

NuoH
MSWISPELIEILLTILKAVVILLVVVTCGAFMSFGERRLLGLFQNRYGPNRVGWGGS
LQLVADMIKMFFKEDWIPKFSDRVIFTLAPMIAFTSLLLAFAIVPVSPGWVVADLNIGI
LFFLMMAGLAVYAVLFAGWSSNNKYSLLGAMRASAQTLSYEVFLGLSLMGVVAQA
GSFNMTDIVNSQAHVWNVIPQFFGFITFAIAGVAVCHRHPFDQPEAEQELADGYHI
EYSGMKFGLFFVGEYIGIVTISALMVTLFFGGWQGPLLPPFIWFALKTAFFMMMFIL
IRASLPRPRYDQVMSFGWKICLPLTLINLLVTAAVILWQAQ*

NuoI
MTLKELLVGFGTQVRSIWMIGLHAFAKRETRMYPEEPVYLPPRYRGRIVLTRDPD
GEERCVACNLCAVACPVGCISLQKAETKDGRWYPEFFRINFSRCIFCGLCEEACP
TTAIQLTPDFEMGEYKRQDLVYEKEDLLISGPGKYPEYNFYRMAGMAIDGKDKGE
AENEAKPIDVKSLLP*

NuoJ
MEFAFYICGLIAILATLRVITHTNPVHALLYLIISLLAISGVFFSLGAYFAGALEIIVYAGA
IMVLFVFVVMMLNLGGSEIEQERQWLKPQVWIGPAILSAIMLVVIVYAILGVNDQGI
DGTPISAKAVGITLFGPYVLAVELASMLLLAGLVVAFHVGREERAGEVLSNRKDDS
AKRKTEEHA*

NuoK
MIPLQHGLILAAILFVLGLTGLVIRRNLLFMLIGLEIMINASALAFVVAGSYWGQTDG
QVMYILAISLAAAEASIGLALLLQLHRRRQNLNIDSVSEMRG*

NuoL
MNMLALTIILPLIGFVLLAFSRGRWSENVSAIVGVGSVGLAALVTAFIGVDFFANGE
QTYSQPLWTWMSVGDFNIGFNLVLDGLSLTMLSVVTGVGFLIHMYASWYMRGEE
GYSRFFAYTNLFIASMVVLVLADNLLLMYLGWEGVGLCSYLLIGFYYTDPKNGAAA
MKAFVVTRVGDVFLAFALFILYNELGTLNFREMVELAPAHFADGNNMLMWATLMLL
GGAVGKSAQLPLQTWLADAMAGPTPVSALIHAATMVTAGVYLIARTHGLFLMTPE
VLHLVGIVGAVTLLLAGFAALVQTDIKRVLAYSTMSQIGYMFLALGVQAWDAAIFHL
MTHAFFKALLFLASGSVILACHHEQNIFKMGGLRKSIPLVYLCFLVGGAALSALPLV
TAGFFSKDEILAGAMANGHINLMVAGLVGAFMTSLYTFRMIFIVFHGKEQIHAHAVK
GVTHSLPLIVLLILSTFVGALIVPPLQGVLPQTTELAHGSMLTLEITSGVVAVVGILLA
AWLWLGKRTLVTSIANSAPGRLLGTWWYNAWGFDWLYDKVFVKPFLGIAWLLKR
DPLNSMMNIPAVLSRFAGKGLLLSENGYLRWYVASMSIGAVVVLALLMVLR*

NuoM
MLLPWLILIPFIGGFLCWQTERFGVKVPRWIALITMGLTLALSLQLWLQGGYSLTQS
AGIPQWQSEFDMPWIPRFGISIHLAIDGLSLLMVVLTGLLGVLAVLCSWKEIEKYQG
FFHLNLMWILGGVIGVFLAIDMFLFFFFWEMMLVPMYFLIALWGHKASDGKTRITAA
TKFFIYTQASGLVMLIAILALVFVHYNATGVWTFNYEELLNTPMSSGVEYLLMLGFFI
AFAVKMPVVPLHGWLPDAHSQAPTAGSVDLAGILLKTAAYGLLRFSLPLFPNASAE
FAPIAMWLGVIGIFYGAWMAFAQTDIKRLIAYTSVSHMGFVLIAIYTGSQLAYQGAVI
QMIAHGLSAAGLFILCGQLYERIHTRDMRMMGGLWSKMKWLPALSLFFAVATLGM
PGTGNFVGEFMILFGSFQVVPVITVISTFGLVFASVYSLAMLHRAYFGKAKSQIASQ
ELPGMSLRELFMILLLVVLLVLLGFYPQPILDTSHSAIGNIQQWFVNSVTTTRP*

NuoN
MTITPQNLIALLPLLIVGLTVVVVMLSIAWRRNHFLNATLSVIGLNAALVSLWFVGQA
GAMDVTPLMRVDGFAMLYTGLVLLASLATCTFAYPWLEGYNDNKDEFYLLVLIAAL
GGILLANANHLASLFLGIELISLPLFGLVGYAFRQKRSLEASIKYTILSAAASSFLLFG
MALVYAQSGDLSFVALGKNLGDGMLNEPLLLAGFGLMIVGLGFKLSLVPFHLWTP
DVYQGAPAPVSTFLATASKIAIFGVVMRLFLYAPVGDSEAIRVVLAIIAFASIIFGNLM
ALSQTNIKRLLGYSSISHLGYLLVALIALQTGEMSMEAVGVYLAGYLFSSLGAFGVV
SLMSSPYRGPDADSLFSYRGLFWHRPILAAVMTVMMLSLAGIPMTLGFIGKFYVLA

	VGVQAHLWWLVGAVVVGSAIGLYYYLRVAVSLYLHAPEQPGRDAPSNWQYSAGG IVVLISALLVLVLGVWPQPLISIVRLAMPLM*

Supplementary Table S3 | PCR cycles parameters.

		pBAD linearisation	pUC19 linearisation	Fragment linearisation	Single point mutation
**Step**		PCR cycle			
**Denaturation**	30 cycles	95°C			
**Annealing**		60°C			
**Elongation**		8 min 45 s	1 min 30 s	45 s	1 min 30 s
**Final extension**		5 min			

Supplementary Table S4 | PCR reaction mixture.

	pBAD linearisation	pUC19 linearisation	Fragment linearisation	Single point mutation
**Polymerase**	**SuperFi II**	**Phusion plus**	**Phusion plus**	**Phusion plus**
5X Buffer	1X			
GC enhancer	1X			
dNTPs	0.2 mM			
Forward primer	0.6 mM	0.5 mM		
Reverse primer	0.6 mM	0.5 mM		
H_2O	Up to 20 µL	Up to 50 µL		
Template	2-10 ng μL^{-1}			
Polymerase	1 U			

Supplementary Table S5 | Composition of overexpression media and purification buffers.

**Autoinduction media**	1% (w/v) peptone, 0.5% (w/v) yeast extract, 0.4% glycerol, 25 mM Na_2HPO_4, 25 mM KH_2PO_4, 50 mM NH_4Cl, 5 mM Na_2SO_4 •$10H_2O$, 2 mM $MgSO_4$•$7H_2O$, 0.2% (w/v) L-arabinose, 0.05% (w/v) glucose, 30 mg L^{-1} Fe-NH_4-citrate, 0.5 mM L-cystein, 50 mg L^{-1} riboflavin
**Cell resuspension buffer**	50 mM MES pH 6.0, 50 mM KCl
**Membrane resuspension buffer**	50 mM MES pH 6.0, 50 mM KCl, 5 mM $MgCl_2$, 10% glycerol
**IMAC buffer A**	50 mM MES pH 6.0, 50 mM KCl, 5 mM $MgCl_2$, 10% glycerol, 0.005% LMNG,<br>20 mM Imidazole
**IMAC Buffer B**	50 mM MES pH 6.0, 50 mM KCl, 5 mM $MgCl_2$, 10% glycerol, 0.005% LMNG,<br>500 mM Imidazole
**SEC Buffer**	50 mM MES pH 6.0, 50 mM KCl, 5 mM $MgCl_2$, 10% glycerol, 0.005% LMNG
